# mTORC2-mediated PDHE1α nuclear translocation links EBV-LMP1 reprogrammed glucose metabolism to cancer metastasis in nasopharyngeal carcinoma

**DOI:** 10.1038/s41388-019-0749-y

**Published:** 2019-02-11

**Authors:** Jun Zhang, Lin Jia, Tengfei Liu, Yim Ling Yip, Wing Chung Tang, Weitao Lin, Wen Deng, Kwok Wai Lo, Chanping You, Maria Li Lung, Hong Lok Lung, Annie Lai-Man Cheung, Sai Wah Tsao, Chi Man Tsang

**Affiliations:** 1 School of Biomedical Sciences, Li Ka Shing Faculty of Medicine, The University of Hong Kong, Hong Kong, China; 2 School of Nursing, Li Ka Shing Faculty of Medicine, The University of Hong Kong, Hong Kong, China; 3 Department of Anatomical & Cellular Pathology, State Key Laboratory of Translational Oncology, The Chinese University of Hong Kong, Hong Kong, China; 4 Department of Clinical Oncology, Li Ka Shing Faculty of Medicine, The University of Hong Kong, Hong Kong, China; 5 Center for Cancer Research, The University of Hong Kong, Hong Kong, China; 6 Department of Biology, Hong Kong Baptist University, Hong Kong, China

**Keywords:** Cancer metabolism, Prognostic markers, Tumour virus infections

## Abstract

EBV infection of preinvasive nasopharyngeal epithelium is believed to be an initiation step during pathogenesis of nasopharyngeal carcinoma (NPC), but the mechanisms remain poorly understood. Here we report a novel mechanism driving NPC metastasis through the EBV-encoded LMP1-mediated metabolic reprogramming, via activation of IGF1-mTORC2 signaling and nuclear acetylation of the *Snail* promoter by the PDHE1α, an enzyme involved in glucose metabolism. Mechanistically, EBV-LMP1 increases the cellular secretion of IGF1 which promotes phosphorylation of IGF1R to activate mTORC2/AKT signaling linking glucose metabolism to cell motility. LMP1 expression facilitates translocation of mitochondrial PDHE1α into the nucleus in a phosphorylation-dependent manner at Ser^293^ residue. Functionally, nuclear PDHE1α promotes H3K9 acetylation on the *Snail* promoter to enhance cell motility, thereby driving cancer metastasis. Importantly, the IGF1/mTORC2/PDHE1α/Snail axis correlates significantly with disease progression and poor prognosis in NPC patients. This study highlights the functional importance of IGF1-mTORC2-PDHE1α signaling mediated by EBV-LMP1 in NPC pathogenesis.

## Introduction

Oncogenic viruses commonly interfere with the host metabolic signaling pathways to exert their transformation properties [1]. Epstein-Barr virus (EBV) is a human γ-herpesvirus closely associated with both lymphoid [2] and epithelial malignancies including nasopharyngeal carcinoma (NPC) [3, 4] and gastric cancer [5]. EBV-associated NPC is a special type of head and neck cancer, which is highly invasive and metastatic [6]. EBV infection in NPC is predominantly latent in nature with restricted expression of viral proteins notably the latent membrane protein-1 (LMP1) [3, 4]. *LMP1* is a well-characterized oncogene encoded by EBV and has been postulated to play an essential role in NPC pathogenesis [7, 8]. The roles of LMP1 in glycolysis addiction, a common hallmark of cancer, is emerging as an important mediator in NPC pathogenesis and progression [9–13]. The role of EBV-LMP1 in modulating metabolic pathways to promote dissemination of tumor cells has not been previously reported.

Tumor metastasis is a major cause of treatment failure [14]. Epithelial-mesenchymal transition (EMT) is an essential process in tumor metastasis. The involvement of *Snail* in EMT is well documented. Expression of *Snail* enhanced cell motility and invasiveness by downregulating epithelial markers and upregulating mesenchymal markers [15]. Invasive cancer cells undergo metabolic reprogramming to facilitate their dissociation from primary site and migration to distant metastatic sites [16]. Transformation of cells from a preinvasive stage to highly invasive state often exhibits increased glycolysis to generate energy for enhanced cell motility [17]. Increasing evidences suggested that some of the core regulators of metabolism, such as PKM2 and PGAM1, are involved in cancer metastasis [18, 19]. Investigation into the interplay between cancer metabolism and cell motility may provide novel targets to suppress cancer metastasis.

Activation of mTORC2 by growth factors is specifically evidenced by AKT phosphorylation at the Ser^473^ site [20]. The mTORC2 could regulate glycolytic enzymes by post-translational modification, for example, phosphorylation of pyruvate dehydrogenase kinase 1 (PDHK1) on Thr^346^, which further phosphorylates and inactivates the substrate pyruvate dehydrogenase complex (PDC) [21]. The PDC normally resides in the mitochondria and is responsible for converting the pyruvate to acetyl-coA. In normal cells, the acetyl-coA molecule is largely oxidized through the tricarboxylic acid (TCA) cycle for energy synthesis. Recent studies have reported that accumulation of PDC in nucleus modulates histone acetylation and induces epigenetic modification to support cell cycle progression [22, 23].

In this study, we dissected how EBV-LMP1 reprograms glucose metabolism to enhance cell motility. A novel signaling axis of LMP1 to drive cell motility was observed involving enhanced secretion of insulin-like growth factor 1 (IGF1) to activate mTORC2/AKT pathway, which facilitates nuclear translocation of PDHE1α, thereby driving histone H3K9 acetylation, eventually leading to the activation of the *Snail* promoter. This signaling axis also potentiates metastasis of NPC cells in vivo and has clinical implication on prognosis of NPC patients.

## Results

### EBV infection induces glycolytic addiction in nasopharyngeal epithelial cells

**Fig. 1:**
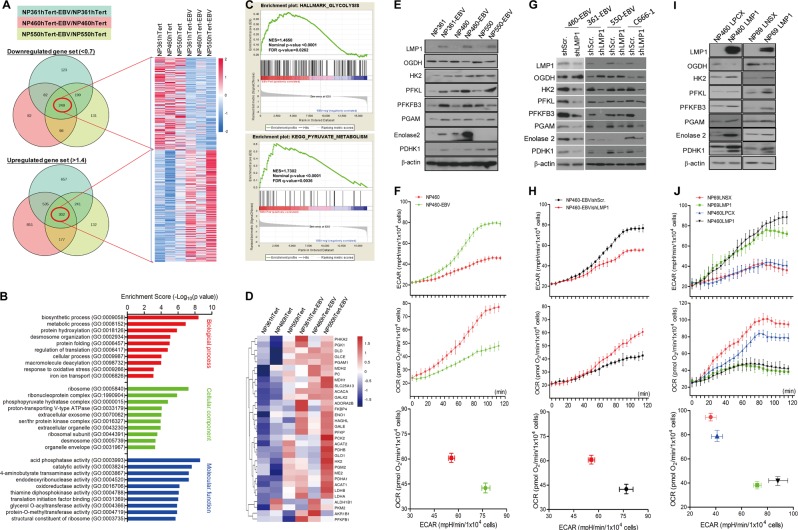
EBV infection induces glycolytic addiction in nasopharyngeal epithelial (NPE) cells. **a** Venn diagram describing the distribution of DEG after EBV infection compared with parental controls in NP361hTert, NP460hTert, and NP550hTert. The numbers in red circle denote the number of overlapping genes, which are either 1.4 times over- (lower panel) or 0.7 times under-expressed (upper panel) in all the three cell lines. The heatmap represents the normalized gene sets of downregulated and upregulated genes. **b** The GO analysis for the DEG in **a** according to the biological process, cellular component, and molecular function, respectively. **c** GSEA showing the gene sets of glycolysis and pyruvate metabolism were upregulated in cells with EBV infection. In each panel, the top portions of the plots show the running enrichment score (ES) for the gene set. Each vertical bar in the middle portions represents a gene, and genes enriched in either condition are at the right (EBV-positive) or left (EBV-negative) parts of the graph. The normalized enrichment score (NES), the *p* value, and the false discovery rate (*q*-value) are indicated in the insert. **d** Heatmap showing renormalizing genes of the key enzymes involved in glycolysis and pyruvate metabolic process. **e**, **f** The EBV-infected NPE cells and the control cells, **g**, **h** EBV-positive NPE cells infected with shScr. and shLMP1, and **i**, **j** NP69 and NP460 cells with stable expression of LMP1 were analyzed by western blotting for detecting the expression of metabolism-associated enzymes using specific antibodies (**e**, **g**, **i**). β-actin expression was used as the loading control. **f**, **h**, **j** ECAR and OCR were measured in indicated cell lines simultaneously by using the 96-well plate reading system (Victor, PerkinElmer) in real time. Cells were plated at 10,000 cells/well for 24 h, then the cells were incubated with the ECAR or OCR reagents according to the manual

Infection of EBV in three hTERT-immortalized nasopharyngeal epithelial (NPE) cells was confirmed by expression of green fluorescent protein tagged to EBV genome and detection of EBV-DNA fluorescence in situ hybridization (Fig. S1A). Expression of latent EBV genes (*EBER*, *EBNA1*, *LMP1*, and *LMP2A*) in infected NPE cells was detected by western blotting and reverse transcription-PCR (Fig. S1B–C). The differentially expressed genes (DEG) of EBV-infected and -uninfected cells were shown in the Venn diagrams after normalization revealing 249 downregulated genes and 302 upregulated genes (Fig. 1a). A heatmap representing these two groups of DEGs was generated (Fig. 1a; specific gene names and functions are shown in Table S1). We further performed the enrichment analysis of these DEGs based on the Gene Ontology Consortium (GO) database [24] and observed a large population of genes are involved in cell metabolism (Fig. 1b). Using gene set enrichment analysis (GSEA) [15], we observed that genes involved in glycolysis and pyruvate metabolism were statistically enriched in EBV-infected cells supporting a role of EBV infection in metabolic reprogramming (Fig. 1c; Fig. S2A–B). A heatmap generated with the key enzymes involved in glycolysis and pyruvate metabolic process also indicated their upregulation in EBV-infected cells (Fig. 1d). Upregulation of glycolysis enzymes and downregulation of oxidative phosphorylation enzymes in EBV-infected cells were confirmed using western blot (Fig. 1e) and RT-PCR (Fig. S2C). We also detected an enhanced glycolysis phenotype in EBV-infected cells by comparing their extracellular acidification ratio (ECAR) and oxygen consumption ratio (OCR) (Fig. 1f; Fig. S2E). Furthermore, EBV-infected cells were more sensitive to 2-deoxy-d-glucose (2-DG; a glucose analog used as a glycolytic inhibitor) while EBV-uninfected cells were more sensitive to oligomycin (a mitochondrial ATP synthesis inhibitor) (Fig. S2D) providing further evidence that EBV-infected cells were addictive to glycolysis. These results support a reprograming of glucose metabolism in EBV-infected NPE cells from oxidative phosphorylation (OXPHOS) to aerobic glycolysis.

### EBV-LMP1 is involved in reprogramming the glucose metabolism

We further confirmed the involvement of LMP1 in conferring the altered metabolic phenotype by suppressing LMP1 expression by shLMP1 (Fig. S2F). Suppression of LMP1 resulted in downregulation of key glycolytic genes (Fig. S2G) and proteins (Fig. 1g). We also observed a higher lactate production and lower oxygen consumption in control cells compared with the shLMP1-transfected cells (Fig. 1h and Fig. S2I). Furthermore, suppression of LMP1 also reduced sensitivity to 2-DG and enhanced sensitivity to oligomycin (Fig. S2H).

Both western blot (Fig. 1i) and RT-PCR (Fig. S2J) also confirmed that LMP1 enhanced the expression of glycolytic enzymes but reduced the OXPHO enzymes. An enhanced glycolytic phenotype was also observed in LMP1-expressing NPE cell lines, which produced more lactate but consumed less oxygen compared to control cells (Fig. 1j). Furthermore, LMP1-expressing cells were more sensitive to 2-DG but less sensitive to oligomycin confirming that LMP1-expressing cells were more dependent on glycolysis (Fig. S2K). All these findings support the involvement of LMP1 in mediating EBV-driven metabolic reprogramming.

### Glycolysis is involved in LMP1-enhanced cell motility in NPE cells

**Fig. 2:**
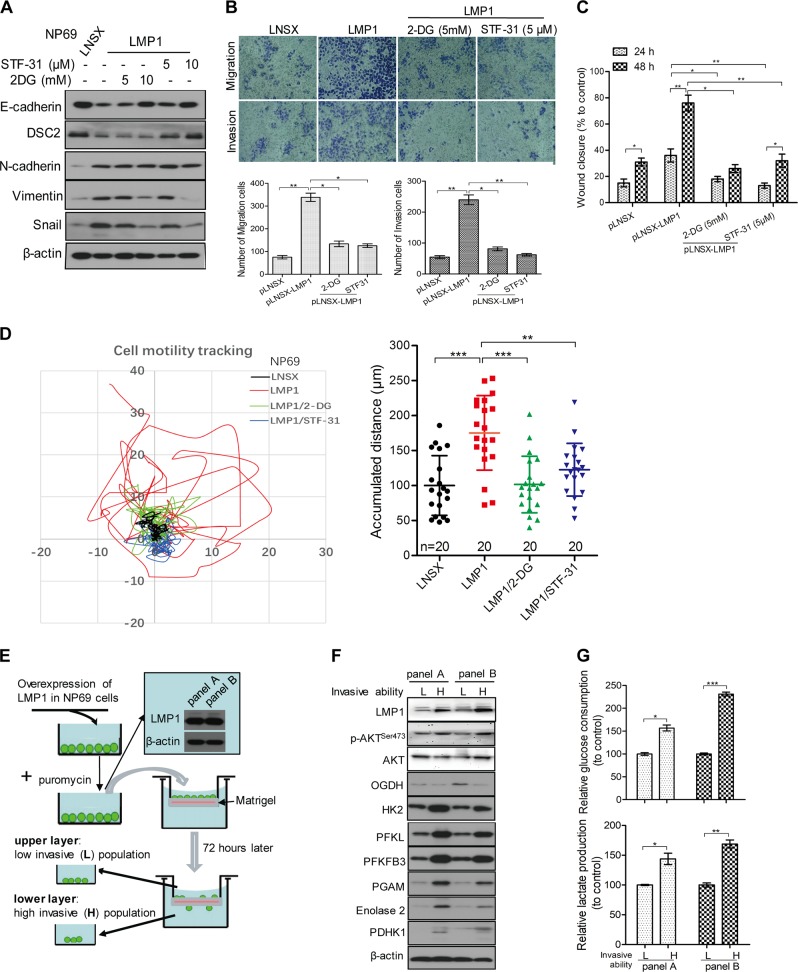
Glycolysis is involved in LMP1-enhanced cell motility in NPE cells. **a** NP69-LNSX and -LMP1 cells were stimulated with different doses of 2-DG or STF-31 for 48 h. The cells were then lysed, and the lysates were analyzed by western blotting for detecting the expression of cell motility-associated molecules using specific antibodies. β-actin expression was used as the loading control. **b** Cells were treated with the indicated small molecules, then cell migration and invasion were assayed using uncoated Millipore Transwell chambers or coated ones with Matrigel respectively. Cells that could transmigrate through the membrane were stained and representative images are shown. The number of cells in five random microscopic fields was counted for each group. **c** Cells were treated with the indicated small molecules and then the confluent monolayer cells were scraped. The migration into the wounded area was assessed 24 and 48 h after scraping and the wound closure was statistically analyzed. **d** NP69 cells with stable expression of LMP1 or control vector were seeded on the coverglass chamber. After attachment, the cells were treated with 2-DG (5 mM) and STF-31 (5 μM), then cells were observed under time-lapse microscope. Representative tracks of cell movement were traced and visualized using metaphase software every 10 min for 24 h. The accumulated distance was analyzed by metaphase software. **e** Schematic diagram to show the isolation of two populations of cells with different degree of invasive ability, two independent experiments were indicated as panel **a** and panel **b**. **f** Two populations of cells isolated in **e** were lysed for western blotting analysis using the indicated antibodies. β-actin expression was used as the loading control. **g** The glucose consumption and lactate production of the two populations of cells with different invasive potentials were determined. Data are means ± SD (means ± SEM for **d**). *p < 0.05; **p < 0.01; ***p < 0.005

LMP1 has been reported to induce EMT and metastatic properties in NPC [25, 26]. Our study confirmed that LMP1 expression induces EMT in NPE cells (Fig. 2a). To investigate the involvement of glycolysis in cell motility, we treated LMP1-expressing cells with glycolytic inhibitors, 2-DG and STF-31 (a selective inhibitor for the glucose transporter-1). These chemicals effectively attenuated LMP1-enhanced EMT in a dose-dependent manner (Fig. 2a), suppressed migration and invasion (Fig. 2b), and wound healing (Fig. 2c and Fig. S3A). Live-cell tracking over 24 h period also revealed suppression of LMP1-enhanced cell migration in the presence of glycolytic inhibitors (Fig. 2d and Movie S1–4). We then separated the LMP1-overexpressing NP69 cells into high- and low-invasive populations by plating them in an invasion chamber coated with Matrigel. High-invasive cell population (which migrated through the invasion chamber) and low-invasive cell population (which remained in the upper chamber) were obtained (Fig. 2e). Western blot analysis revealed higher expression of glycolytic enzymes in the high-invasive cell populations compared to the low-invasive cell populations (Fig. 2f). A higher rate of glucose uptake and lactate production was also observed in the high-invasive cell population implicating the involvement of glycolysis in mediating LMP1-enhanced cell motility (Fig. 2g).

### LMP1 activation of mTORC2 by autocrine secretion of IGF1 links EBV-reprogrammed glucose metabolism to cell motility

**Fig. 3:**
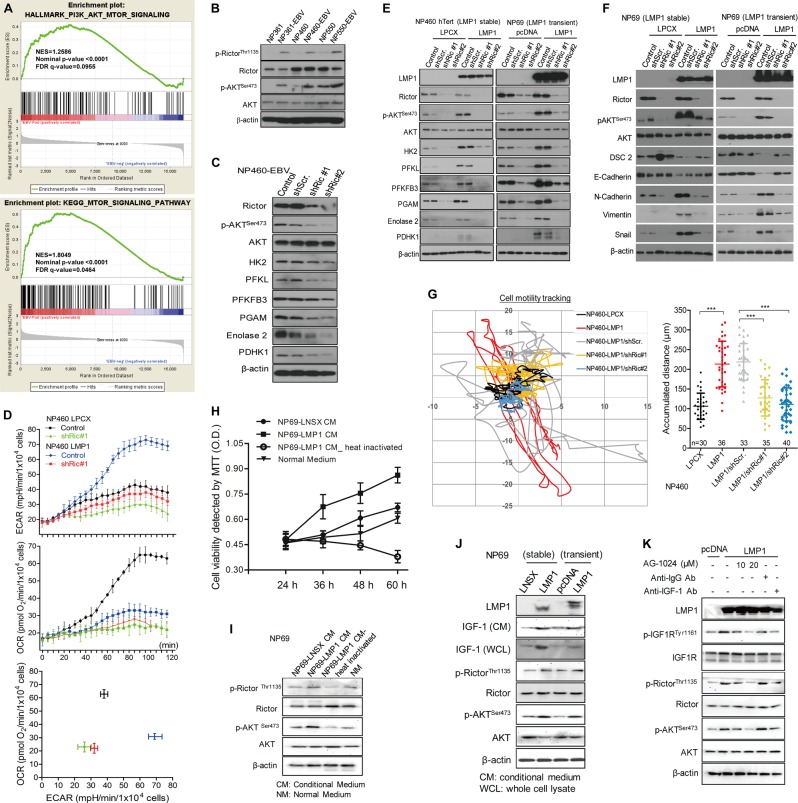
LMP1 activation of mTORC2 by autocrine secretion of IGF1 links Epstein-Barr virus (EBV)-reprogrammed glucose metabolism to cell motility. **a** GSEA showing the EBV infection upregulate PI3K-AKT-mTOR signaling-dependent gene sets. In each panel, the top portions of the plots show the ES for the gene set. Each vertical bar in the middle portions represents a gene, and genes enriched in either condition are at the right (EBV-positive) or left (EBV-negative) parts of the graph. The NES, the *p* value, and the false discovery rate (*q*-value) are indicated in the insert. **b** Different NPE cell lines with or without EBV infection, and **c** NP460-EBV cell infected with shRictor or control empty lentiviral vector were subjected to western blotting for detecting the expression of mTORC2 activity and metabolism-associated molecules using specific antibodies. β-actin expression was used as the loading control. **d** NP460-LPCX and NP460-LMP1 cells infected with shRictor or control empty lentiviral vector were plated at 10,000 cells/well for 24 h, then the cells were incubated with the ECAR or OCR reagents according to the manual. ECAR and OCR were measured simultaneously by using the 96-well plate reading system (Victor, PerkinElmer) in real time. **e**, **f** NPE cells with stable or transient overexpression of LMP1 were infected with shRictor or control vector. Then, cells were lysed and analyzed by western blotting. **g** shRictor- or its control vector-infected NP460-LPCX and LMP1 cells were seeded on the coverglass chamber and observed under time-lapse microscope. Representative tracks of cell movements were traced and visualized using metaphase software every 10 min for 24 h. The accumulated distance was analyzed by metaphase software. **h** Cell viability of NP69 was measured by MTT assay after incubating with different conditional medium for the indicated times. **i** The NP69 cells were incubated with the different conditional medium for 48 h, followed by western blotting for detection of mTORC2 signaling activity using the indicated antibodies. **j** After culturing different pairs of NPE and NPC cells for 48 h, the culture media as well as the cell lysates were subjected to western blot assay using the indicated antibodies. **k** NP69-pLNSX and LMP1 cells were treated with different doses of AG-1024 and antibodies against-IgG or IGF1 for indicated time, followed by western blot assay using the indicated antibodies. Data are means ± SD (means ± SEM for **g**). ***p < 0.005

Our RNA-sequencing data showed enrichment of genes in PI3K/AKT as well as mTOR signaling in EBV-infected NPE cells (Fig. 3a; Fig. S4A–B). We have previously reported that LMP1 activates mTORC1 to enhance glycolysis [9]. Here we report that mTORC2 was activated in EBV-infected NPE cells (Fig. 3b). Knockdown of Rictor not only abrogated phosphorylation of AKT at Ser^473^ and downregulated multiple glycolytic enzymes (Fig. 3c) but also lowered the rates of glycolysis (Fig. 3d, e). Suppression of glycolysis was observed after blocking AKT activation (Fig. S5A–C). All these supported a role of mTORC2, in addition to mTORC1, in mediating LMP1-induced glycolysis.

We further demonstrated that inhibition of mTORC2/AKT signaling could reverse LMP1-induced EMT, as indicated by the downregulation of mesenchymal markers but upregulation of epithelial markers (Fig. 3f and Fig. S5B). Suppression of mTORC2/AKT signaling also attenuated LMP1-induced cell motility (Fig. 3g and Movie S5–9) and invasive properties (Fig. S4C–E, and S5D−G). Our supplementary results using gene knockdown and inhibitor approaches supported a more prominent role of mTORC2 in LMP1-enhanced cell motility (Fig. S4C).

We further investigated the involvement of autocrine mechanisms in mTORC2 signaling activation by LMP1 (Fig. S6A). Both cell proliferation and mTORC2 activation in parental cells were enhanced after incubating with conditioned medium harvested from LMP1-expressing cells (Fig. 3h, i). Previous study had reported that LMP1 promotes IGF1 expression to drive cell proliferation [27], which was confirmed in immortalized NPE cell (Fig. S6B). Increased expression of IGF1 was associated with activation of mTORC2 in LMP1-expressing NP69 cells (Fig. 3j). Treatment with neutralizing antibody against IGF1 or IGF1R inhibitor AG-1024 suppressed LMP1-induced mTORC2 activation (Fig. 3k) and its downstream events including cell proliferation (Fig. S6C), glucose metabolism (Fig. S6D&E), and cell motility (Fig. S6F&G). These results support an important role of LMP1/IGF1/IGF1R signaling axis to activate mTORC2 and its downstream events.

### Nuclear localization of PDHE1α is involved in mediating LMP1-enhanced motility

**Fig. 4:**
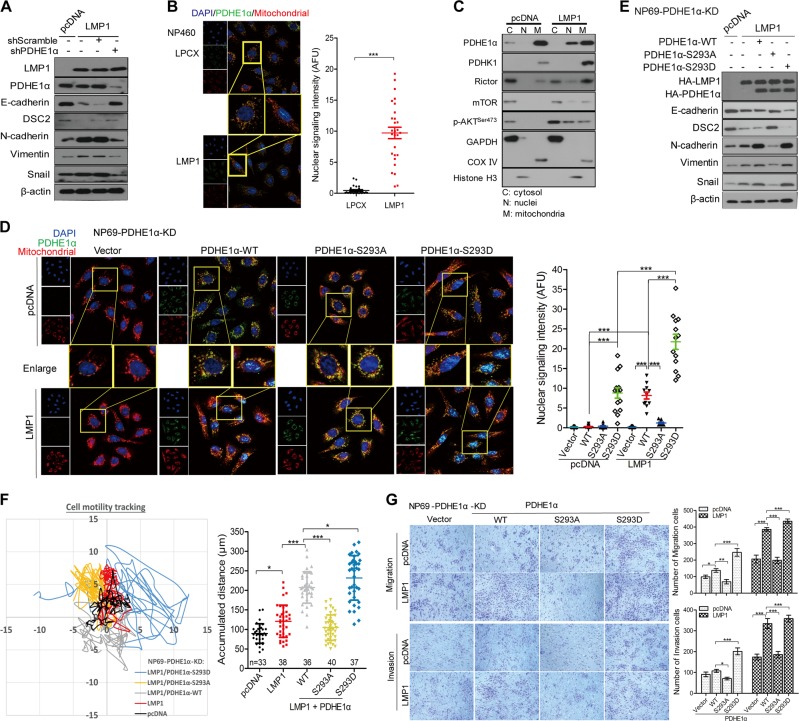
Nuclear translocation of PDHE1α is involved in mediating LMP1-enhanced EMT and motility in NPE cells. **a** NP69 cells with overexpression of LMP1 were infected with shPDHE1α or control vector for 48 h, cells were lysed, and subjected to western blotting for detecting the expression of cell motility-associated proteins. **b** Cells were co-stained with MitoTracker Red (mitochondrial marker), PDHE1α (green), and Hoechst33258, and representative images were taken using a Zeiss LSM800 system. The nuclear signaling intensity was analyzed and is shown in histogram. **c** Subcellular fractionations of NP69 and NP69-LMP1 were isolated for demonstrating the localization of indicated proteins in various subcellular compartments. GAPDH, COX IV, and Histone H3 were used as cytosol, mitochondrial, and nucleus markers respectively. **d** NP69-PDHE1α-KD cells were first stably reconstituted with either wild-type (WT) or mutant PDHE1α, and then transfected with pcDNA or pcDNA-LMP1, followed by co-staining with MitoTracker Red (mitochondrial marker), PDHE1α (green), and Hoechst33258. Representative images were taken using a Zeiss LSM800 system. **e** Indicated cells were also analyzed by western blotting for detecting the expression of cell motility-associated markers. **f** Cells were seeded on the coverglass chamber and observed under time-lapse microscope. Representative tracks of cell movements that were traced and visualized using metaphase software every 10 min for 24 h. The accumulated distance was analyzed by metaphase software. **g** Cell migration and invasion were assayed using uncoated Millipore Transwell chambers and coated ones with Matrigel respectively. Representative images of cells at the bottom surface are shown. The number of cells in five random fields were counted for each group. Data are means ± SD (means ± SEM for **b**, **d**, **f**). *p < 0.05; **p < 0.01; ***p < 0.005

Pyruvate can be converted to acetyl-CoA in OXPHOS by the pyruvate dehydrogenase (PDH) complex in normal cells. Notably, our heatmap summarizing the changed expression of glycolysis enzymes including PDHA1 (also known as PDHE1α) (Fig. 1d). We have investigated the role of PDHE1α in LMP1-mediated cell motility. Knocking down PDHE1α suppressed the LMP1-enhanced EMT (Fig. 4a). Interestingly, immunofluorescence staining revealed an increase of PDHE1α in nucleus but a decrease in mitochondrion suggesting a nuclear translocation of PDHE1α from mitochondria to nucleus in LMP1-expressing cells (Fig. 4b). We further confirmed the nuclear translocation of PDHE1α by western blot analysis of cytosolic (C), nuclear (N), and mitochondrial (M) subcellular fractions (Fig. 4c). In addition, knockdown of Rictor suppressed nuclear levels of PDHE1α (Fig. S7A), which implicates that LMP1 activation of mTORC2 is involved in regulating the nuclear accumulation of PDHE1α. Phosphorylation of PDHE1α at Ser^293^ by its upstream kinase, PDHK1, is a common event in cancer cells [28]. We further showed that blocking the PDHK1 activation by expression of its dominant negative mutant (PDHK1-T346A) suppressed the LMP1-mediated nuclear accumulation of PDHE1α (Fig. S7B). The role of phosphorylation of Ser^293^ of PDHE1α in its nuclear accumulation was shown by the following experiments. The endogenous PDHE1α was first knocked down by short hairpin RNA (referred as NP69-PDHE1α-KD cells), then followed by either overexpression of the wild-type (WT) construct of PDHE1α (PDHE1α-WT) or the constitutive activation of phosphomimic PDHE1α construct (PDHE1α-S293D). Reconstitutive expression of the WT or S293D-PDHE1α constructs, but not with the phosphorylation-dead PDHE1α construct (PDHE1α-S293A), significantly enhanced nuclear localization of PDHE1α (Fig. 4d). Moreover, the EMT markers, accumulated cell motility distance as well as migration and invasion properties were also restored after reconstitutive expression of PDHE1α or S293D-PDHE1α (Fig. 4e–g). Taken together, these results support that Ser^293^ phosphorylation of PDHE1α is responsible for its nuclear localization to mediate the LMP1-enhanced cell motility.

### Nuclear PDHE1α promotes histone acetylation of *Snail* promoter to mediate LMP1-enhanced cell motility

**Fig. 5:**
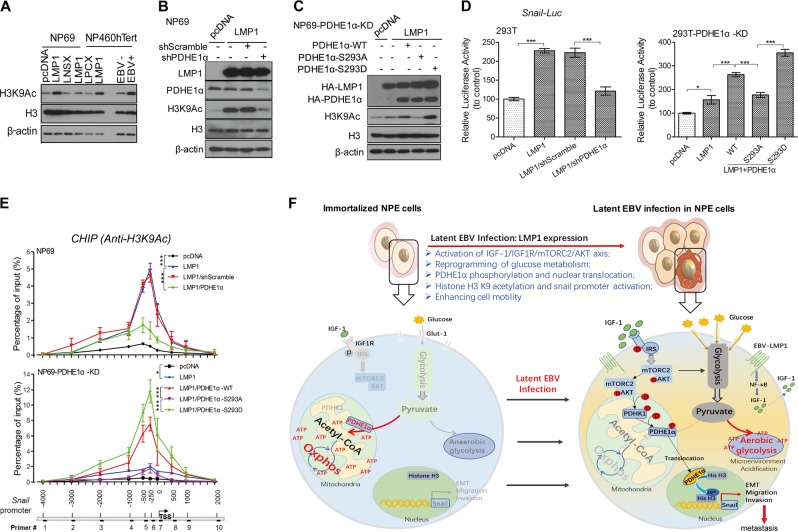
Nuclear PDHE1α promotes histone acetylation at *Snail* promoter to mediate LMP1-enhanced cell motility. **a** NP69 and NP460 cells with overexpression of LMP1 or stable infection of EBV were subjected to western blotting for analyzing the level of histone acetylation using the indicated antibodies. **b** NP69 cells were transfected with pcDNA or LMP1 constructs and then infected with shPDHE1α for 48 h, cells were lysed and subjected to western blotting for analysis using the indicated antibodies. **c** NP69 cells with stable knockdown of PDHE1α (NP69-PDHE1α-KD) were co-transfected with pcDNA or LMP1 as well as different PDHE1α constructs, after 48 h cells were lysed and subjected to western blotting for analysis using the indicated antibodies. β-actin expression was used as the loading control. **d** The lysates of the following cell lines were subjected to luciferase reporter assay. Left panel: 293T-pcDNA or 293T-LMP1 cells were transfected with shScramble and shPDHE1α; right panel: 293T cells with stably knockdown of PDHE1α (293T-PDHE1α-KD) were first transfected with either pcDNA or LMP1 and then infected with lentivirus with wild-type (WT) or mutant PDHE1α. **e** NP69 and NP69-PDHE1α-KD cells were transfected with pcDNA and LMP1, or/and other knockdown or expression plasmids in various settings as indicated. The cell lysates were subjected to chromatin immunoprecipitation (ChIP) assay using an anti-H3K9Ac antibody. PCR was performed to amplify the different regions indicated in the lower panel. **f** Schematic diagram of LMP1-induced IGF1/mTORC2/PDHE1α/H3K9Ac axis links glucose metabolism to cell motility and drives NPC pathogenesis. In immortalized nasopharyngeal epithelial cell, cells metabolize glucose through oxidative phosphorylation to produce ATP. After latent EBV infection, the EBV-LMP1 expression reprograms glucose metabolism from oxidative phosphorylation to aerobic glycolysis, which is an essential process for promoting cell motility. Data are means ± SD. *p < 0.05; ***p < 0.005

Nuclear PDHE1α has recently been reported to promote histone acetylation to control cell cycle progression [22, 23]. Interestingly, expression of LMP1 as well as EBV infection also elevated the H3K9 acetylation (Fig. 5a). PDHE1α knockdown significantly suppressed LMP1-induced H3K9 acetylation (Fig. 5b). The LMP1-mediated H3K9 acetylation in NP69-PDHE1α-KD cells was restored by expression of the WT- or S293D-PDHE1α constructs but not S293A-PDHE1α construct (Fig. 5c). These findings support a role of nuclear translocated PDHE1α in LMP1-associated epigenetic modification. The Snail expression has profound effects on EMT in NPC [29]. We observed that activation of the *Snail* promoter by LMP1 could be suppressed by knocking down PDHE1α in 293T cells (Fig. 5d). We further showed that expression of the WT- and S293D-PDHE1α constructs, but not S293A-PDHE1α construct could reconstitute activation of *Snail* promoter by LMP1 (Fig. 5d). Similarly, LMP1-induced H3K9 acetylation and *Snail* promoter activation could be suppressed by inhibition of mTORC2/AKT/PDHK1 signaling activation (Fig. S8A–D). These results provide evidences that nuclear translocation of PDHE1α facilitates H3K9 acetylation, which modulates *Snail* expression to mediate cell motility.

We next performed chromatin immunoprecipitation (ChIP)-PCR assay using acetylated H3K9 antibody to validate our observations. The genomic DNA binding to acetyl-H3K9 was amplified by 10 sets of PCR primers covering the neighboring regions of the *Snail* promoter (Fig. 5e, lower panel). Strong amplification signals were detected from primers located at or around *Snail* transcription start site (−1000 to 500 bp; peaking at −250 bp) in LMP1-expressing cells. Binding of acetyl-H3K9 to the *Snail* promoter was diminished if the nuclear PDHE1α was decreased (Fig. 5e, upper panel). Similarly, reconstitution of WT- or S293D-PDHE1α constructs, but not S293A-PDHE1α construct, significantly enhanced the binding of acetyl-H3K9 to the *Snail* promoter (Fig. 5e, middle panel). Together, these results support a role of nuclear PDHE1α to promote H3K9 acetylation and activation of *Snail* promoter in LMP1-mediated cell motility.

### Nuclear localization of PDHE1α promotes invasion and metastasis of NPC cells

**Fig. 6:**
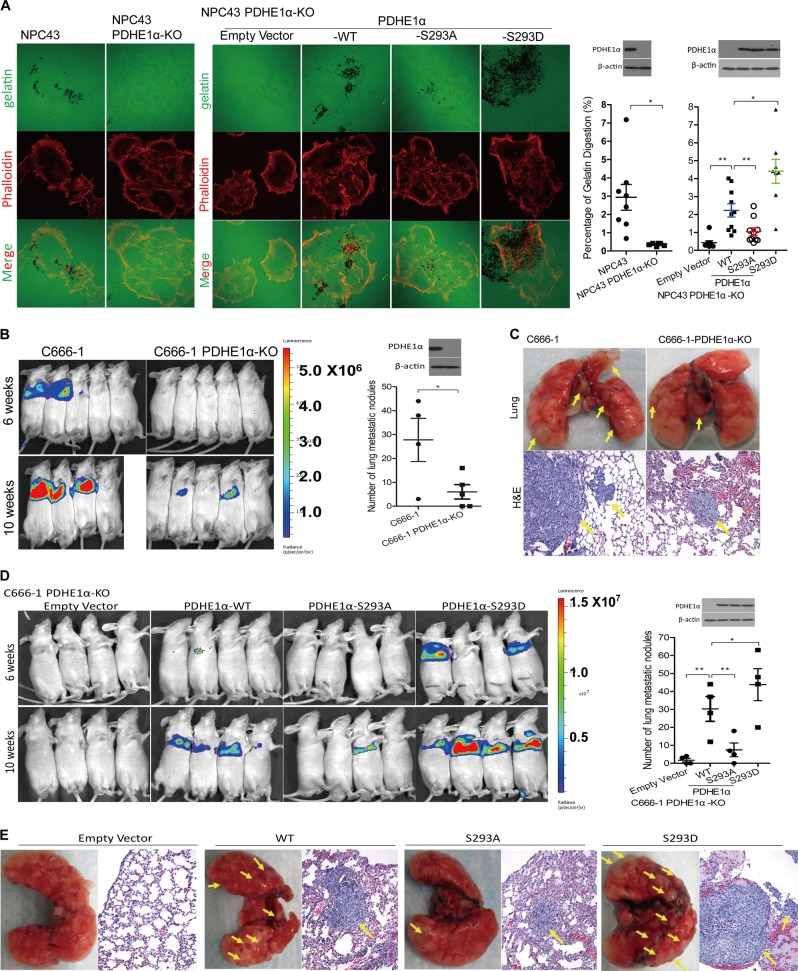
Nuclear translocation of PDHE1α promotes cancer metastasis in nasopharyngeal carcinoma (NPC). **a** NPC43 cells harbored with different PDHE1α mutants were subjected to invadopodia assay by plating on Fluor488-conjuated gelatin. After 24 h, cells were then fixed and stained with Fluo555-Phalloidin. Representative images were captured using a Zeiss LSM800 system. The digestion area of gelatin was quantified by ImageJ software. **b**, **d** C666-1 cells overexpressed with different PDHE1α mutants were cultured and injected into the tail vein of mouse. The luciferase signaling in the lungs indictative for lung metastases was monitored at 6 and 10 weeks after injection, using a PE IVIS Spectrum in vivo imaging system. **c**, **e** The lungs were fixed and stained by hematoxylin and eosin 10 weeks after injection. The lung metastatic tumor nodules per mouse were counted. Data are means ± SEM. *p < 0.05; **p < 0.01

We further examined if PDHE1α nuclear localization is involved in modulation of invasive and metastatic properties of NPC. We used a recently established EBV-positive NPC cell line, NPC43 [30], and examined its invadopodia-dependent ECM degradation ability, which is a common phenotype of metastatic cancer cells. NPC43 cells significantly degraded more gelatin compared with the PDHE1α-KO cells as indicated by the black areas of digested gelatin (Fig. 6a). Next, reconstitution of WT- or S293D-PDHE1α constructs, but not the S293A-PDHE1α constructs in PDHE1α-KO cells could promote the gelatin digestion (Fig. 6a). These observations suggested the involvement of nuclear PDHE1α in mediating invadopodia-dependent ECM degradation.

To further confirm the involvement of PDHE1α in NPC metastasis in vivo, we injected control and PDHE1α-KO C666-1 cells labeled with luciferase through the tail veins of mice and observed the metastasis ability. Growth of C666-1 cells in the lung of injected mice was readily observed 6–10 weeks after injection. In contrast, growth of PDHE1α-KO C666-1 cells was not detected in the lungs of mice 6 weeks after injection and only weak luciferase signals were detected in the lungs of two mice 10 weeks after injection (Fig. 6b). Histological examination of dissected lung confirmed growth of injected C666-1 cells in the lung tissues, but not in mice injected with C666-PDHE1α-KO cells (Fig. 6c). We further observed that reconstitution of WT- or S293D-PDHE1α constructs, but not the S293A-PDHE1α constructs, promoted their growth in the lungs of injected mice (Fig. 6d, e). These results support a role of PDHE1α in promoting NPC metastasis in in vivo model.

### Involvement of IGF1/mTORC2/PDHE1α/Snail axis is associated with NPC progression and poor prognosis in clinical specimens of NPC

**Fig. 7:**
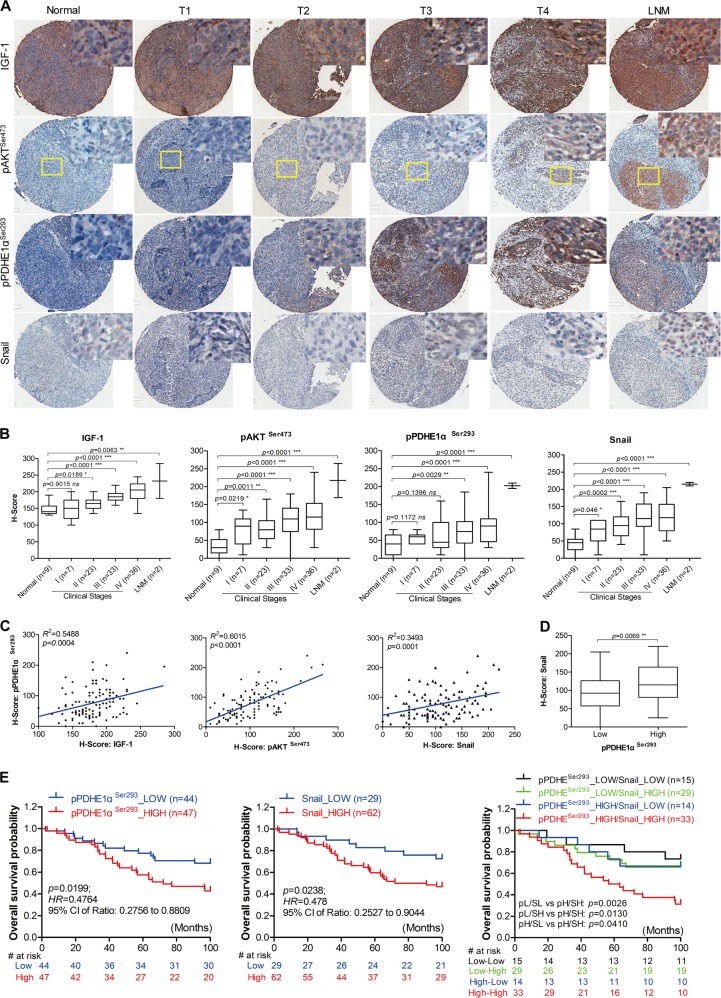
Pathological significance of IGF1/mTORC2/PDHE1α/Snail in NPC patients. **a** Representative immunohistochemical images show IGF1, mTORC2 (pAKT-Ser473), pPDHE1α-Ser293 as well as Snail staining in normal and different clinical stages of NPC tissues. **b** Box whisker analyses showing the correlation of IGF1, mTORC2 (pAKT-Ser473), pPDHE1α-Ser293, and Snail expression with the different clinical stages of NPC samples. The median values of each group are shown by horizontal lines. **c** Dot plot showing the correlation of pPDHE1α-Ser293 expression with the IGF1, mTORC2 (pAKT-Ser473), and Snail expression in different NPC samples. The correlation coefficient *r*^2^ and *p* value were obtained from the linear regression analysis. **d** Box whisker analyses showing the immunoactivity scores of Snail staining in NPC tumors with low and high pPDHE1α-Ser293 expression. The median values of each group are shown by horizontal lines. **e** Kaplan-Meier analysis exhibiting the expression of pPDHE1α-Ser293 and Snail and their correlation with the overall survival of 99 cases of NPC patients. Hazard ratio and 95% confidence interval values are shown in the figures. *p*-values were obtained from the log-rank test in each case. Data are means ± SEM. *p < 0.05; **p < 0.01; ***p < 0.005

A cohort of 101 cases of NPC and 9 cases of NPC adjacent normal tissues was examined by immunocytochemistry. The clinico-pathologic variables are summarized in Table 1. The expression of IGF1/mTORC2/PDHE1α/Snail signaling members was determined by immunocytochemistry. Representative immunostainings are shown in Fig. 7a. Significantly increased expression of IGF1, pAKT, pPDHE1α, and Snail was observed in advanced NPC stages (stage II, III, and IV) and their lymph node metastasis, but low or absent in normal nasopharyngeal tissues and stage 1 NPC (Fig. 7b). High expression of pPDHE1α and Snail was significantly associated with advanced N staging (*p* = 0.0389 and 0.0122) and clinical stages (*p* < 0.001 and *p* = 0.0286). The *H*-scoring cohort showed a positive association of high expression of pPDHE1α^Ser293^ in NPC tissue with IGF1 (*p* = 0.0004, *r*^2^ = 0.5488), pAKT^ser473^ (*p* < 0.0001; *r*^2^ = 0.6015), and Snail (*p* = 0.0001, *r*^2^ = 0.3493) (Fig. 7c, d). LMP1 expression also positively correlated with IGF1/AKT. Association of LMP1 expression with pPDHE1α/Snail was not significant (Fig. S9). A lower overall survival rates were observed in patients with high expression of pPDHE1α^Ser293^ (*p* = 0.0199, hazard ratio (HR) = 0.476) and Snail (*p* = 0.0238, HR = 0.478) (Fig. 7e). Similarly, the group with high expression of both pPDHE1α^Ser293^ and Snail had a significantly lower survival rate (Fig. 7e). Taken together, our data supports the clinical relevance of IGF1/mTORC2/PDHE1α/Snail axis in NPC and their clinical application in disease progression and patient prognosis.

**Table 1:** Correlation between expressions of pPDHE1α^Ser293^ and Snail and clinicopathological features of human NPC

Characteristic	All cases	pPDHE1^Ser293^ protein level				Snail protein level			
		Low	High	*χ* ^2^	*p* Value^a^	Low	High	*χ* ^2^	*p* Value^a^
Age (years)				0.0171	0.8959			1.5936	0.2068
≤50	28	13 (46.43%)	15 (53.37%)			12 (42.86%)	16 (57.14%)		
>50	71	34 (47.89%)	37 (52.11%)			21 (29.58%)	50 (70.42%)		
Sex				0.2719	0.6021			2.0842	0.1488
Female	19	8 (42.11%)	11 (57.89%)			9 (43.47%)	10 (52.63%)		
Male	80	39 (48.75%)	41 (51.25%)			24 (30.00%)	56 (70.00%)		
TNM stages									
T stage				5.3532	0.1477			14.3672	***0.0024***
T1	23	10 (43.48%)	13 (56.52%)			13 (56.52%)	10 (43.48%)		
T2	27	17 (62.96%)	10 (37.04%)			12 (44.44%)	15 (55.56%)		
T3	19	10 (52.63%)	9 (47.37%)			5 (26.32%)	12 (63.16%)		
T4	30	10 (33.33%)	20 (66.67%)			3 (10.00%)	27 (90.00%)		
N stage				8.3745	***0.0389***			10.9117	***0.0122***
N0	20	13 (65.00%)	7 (35.00%)			11 (55.00%)	9 (45.00%)		
N1	33	19 (57.58%)	14 (42.42%)			14 (42.42%)	19 (57.58%)		
N2	37	13 (35.14%)	24 (64.86%)			7 (18.92%)	30 (81.08%)		
N3	9	2 (22.22%)	7 (77.78%)			1 (11.11%)	8 (88.89%)		
M stage				2.4311	0.1190			0.1544	0.6944
M0	93	46 (49.46%)	47 (50.54%)			22 (23.66%)	71 (76.34%)		
M1	6	1 (16.67%)	5 (83.33%)			1 (16.67%)	5 (83.33%)		
Clinical stages				21.7219	***<*** ***0.0001***			9.0560	***0.0286***
I	7	5 (71.43%)	2 (28.57%)			4 (57.14%)	3 (42.86%)		
II	23	18 (78.26%)	5 (21.74%)			13 (56.52%)	10 (43.48%)		
III	33	16 (48.48%)	17 (51.52%)			12 (36.36%)	21 (63.64%)		
IV	36	7 (19.44%)	29 (80.56%)			4 (11.11%)	32 (88.89%)		

*NPC* nasopharyngeal carcinoma

^a^Chi-square test; values in bold-italic were defined as statistical with significant differences (*p* < 0.05)

## Discussion

Despite the close association of NPC with EBV, the role of EBV infection in NPC pathogenesis remains poorly defined [3]. In this study, using various EBV-infected NPE cell systems and *bona fide* EBV-positive NPCs, we have demonstrated a pathological role of EBV and its encoded oncogene, *LMP1*, in driving cell motility through metabolic reprogramming. A detailed schematic diagram summarizing our findings is shown in Fig. 5f.

EBV infection is believed to be an early and essential event during NPC pathogenesis [31]. Alteration of molecular pathways in EBV-infected premalignant NPE cells, which facilitates their malignant transformation has been suggested [3, 4]. The switch to aerobic glycolysis is believed to be beneficial to support cell proliferation in cancer by providing essential intermediates [32]. A recent study has reported that growth arrest of EBV-infected B cells could be induced in the presence of shortage of metabolite supply [33]. A role of LMP1 to support aerobic glycolysis has been reported in multiple studies [9, 10, 12, 34]. LMP1-enhanced glycolysis has been shown to enhance malignant properties of NPC cells [9, 10] by development of chemotherapy resistance [12] and immune escape [13]. In this study, we report a novel role of LMP1-induced glycolysis in promoting cell motility through the mTORC2 signaling. The activation of mTORC2 by LMP1 has been mentioned in a recent study, but the underlying mechanism remains to be defined [35]. In this study, we showed that LMP1 activates mTORC2 through an autocrine activation of IGF1R signaling. Blocking the IGF1-IGF1R signaling axis by neutralizing antibody or IGF1R inhibitor suppressed the activation of mTORC2/AKT signaling. Our observations are consistent with the general concept that mTORC2 activation is responsive to growth factors [36]. Elucidation of the underlying signaling axis may contribute to identification of novel therapeutic targets to control NPC progression.

The involvement of metabolic reprogramming in enhancement of cancer cell motility has been implicated [37]. Several core metabolic enzymes, including PGAM and PKM2, have been shown to have physiological functions in cell motility [19, 38]. Our study showed that PDHE1α, which functions as the primary link between glycolysis and TCA cycle, is closely associated with LMP1-induced cell motility. Previous study has reported that translocation of PDHE1α to nucleus promotes acetylation of histones to modulate multiple cellular activities [23]. In this study, we showed that knockdown of PDHE1α or expression of PDHE1α Ser^293^ mutant significantly attenuated LMP1-induced cell motility. We also demonstrated that Ser^293^ phosphorylation of PDHE1α is essential for its nuclear translocation linking LMP1-induced glucose metabolism to cell motility. The mTORC2/AKT signaling has been reported to mediate glycolysis by regulating the phosphorylation and activation of the glycolytic enzyme, PDHK1, in mitochondria [21]. Our study showed that suppression of mTORC2 or inactivation of PDHK1 decreased the nuclear translocation of PDHE1α. All these observations suggest that LMP1-mediated mTORC2/AKT signaling can mediate PDHE1α phosphorylation and nuclear translocation to modulate gene expression, which are novel properties of LMP1.

The involvement of nuclear PDHE1α to activate *Snail* promoter through epigenetic modification to enhance cell motility is another novel finding. Detailed mechanism that regulating the trafficking of PDHE1α remains to be defined. It was reported that Hsp70 could promote nuclear translocation of the PDH complex by competing with PDHK1 for binding to PDHE1 in a growth factor-dependent manner [23, 39]. In this study, we showed that the LMP1 induces secretion of IGF1 to activate mTORC2/AKT to promote phosphorylation and translocation of PDHE1α into the nucleus to induce acetylation of histone H3 at lysine residue 9. By luciferase assay and ChIP assay, H3K9Ac was shown to be recruited to the promoter region of *Snail*, a well-characterized regulator of EMT [40]. Our in vitro and in vivo models also support a role of PDHE1α in facilitating NPC invasion and metastasis [28]. The detailed involvement of PDHE1α remains to be further delineated.

In summary, the current study reveals a novel role of EBV infection in promoting migratory and invasive ability of NPC cells involving perturbed glucose metabolic pathways and nuclear translocation of PDHE1α mediated by LMP1/IGF1/mTORC2 signaling axis and may reveal new and potentially effective therapeutic target in clinical management of NPC.

## Materials and methods

### Cell lines and cell culture

The NP361hTert, NP460hTert, and NP550hTert are telomerase-immortalized nonmalignant human NPE cell lines established by our laboratory [41–43]. They were cultured in a 1:1 ratio of growth factor supplemented DKSFM and EpiLifeTM medium (GIBCO, Life Technologies TM, Grand Island, NY). The immortalized NP69 NPE cell line with high transfection efficiency and low signaling background [44] was cultured in KSFM medium (GIBCO). The 293 cells were cultured in Dulbecco’s modified Eagle’s medium (Sigma) supplemented with 10% (vol/vol) fetal bovine serum (FBS; GIBCO), 100 U/ml of penicillin, and 100 U/ml of streptomycin. C666-1 cells were cultured in RPMI medium with 10% (vol/vol) FBS, and NPC43 cells were cultured in RPMI medium with 10% (vol/vol) FBS and 4 μM Rock inhibitor. The NPE cells were infected with EBV using a previously published protocol [45].

### Chemicals and reagents

The glucose metabolism-associated inhibitor STF-31 (S7931), AKT signaling inhibitor MK2206 (S1078), and IGF1R tyrosine inhibitor AG-1024 (S1234) were purchased from Selleckchem (Houston, TX); and ROCK inhibitor (ALX-270-333-M025) was got from Enzo Life Sciences (USA). LY294002 (440204) and Oligomycin (495455) were purchased from Calbiochem (Germany). 2-DG (D8375) was purchased from Sigma (St. Louis, Missouri, USA). Fluorescein isothiocyanate-gelatin was obtained from Life Technologies (G13187).

### Bioinformatic analysis of the RNA-sequencing data

The libraries were prepared using the Illumina TruSeq mRNA Library Prep Kit according to the manufacturer’s instructions. Sequencing was performed on an Illumina HiSeq2000 sequencing system (Illumina, San Diego, CA, USA). The gene expression ratio between EBV-infected cells and parental cells was calculated based on the fragments per kilobase million value of each gene. The thresholds for DEGs were set as fold-change > 1.4 or fold-change < 0.7. The up- and downregulated genes in each group respectively were subjected to Venn diagram to illustrate the overlapping genes among the three pairs of cell lines used. The overlapping genes were then subjected to GO for enrichment analysis [24]. GSEA was used to characterize the differences in transcriptome profiles of EBV-positive cells compared to the EBV-negative cells in specific signatures [15]. This method considers the full list of genes, ranked by their correlation with phenotype, and calculates an enrichment score to determine whether the analyzed genes were enriched at specific signatures.

### Assays of extracellular acidification and oxygen consumption

The micro-plate reader system (Victor^3^, PerkinElmer) was used to measure OCR and ECAR. Cells were plated in a 96-well plate with a density of 10,000 cells/well and incubated in a 37 °C incubator for 24 h. After the indicated treatment, cells were incubated with ECAR or OCR reagents according to the manufacturer’s recommendations (ab197244 for ECAR and ab197243 for OCR, Abcam, UK). The ECAR and OCR assay signals were collected at 5 min intervals for about 120 min using excitation and emission wavelengths of 380 and 615 nm respectively.

### Cell motility tracking

Cells were seeded in Lab-Tek glass-bottomed eight-chambered slide (155411, Thermo Scientific) and pretreated as indicated. The chambered slide was then placed on the contained stage of microscope with 5% CO_2_ at 37 ℃. Cell motility was monitored with the widefield microscope (Zeiss, Germany). Cell positions were recorded at every 10-min interval over a period of 24 h and then processed with Track Object Tool in Metamorph analysis software.

### Subcellular fractionation

The subcellular fractions were isolated following a previous published protocol [46].

### ChIP assay

ChIP was performed using a ChIP assay kit (520127, Covaris) following a previously described protocol [9]. Briefly, cells were crosslinked and then lysed. Sheared DNA was incubated overnight at 4 ℃ with primary antibodies against H3K9Ac (#9649, CST) or normal rabbit IgG (sc-2027, Santa Cruz), followed by adding 50 μl magnetic bead into the mixture and incubating at 4 ℃ for 2 h. The chromatin was then washed, the crosslinking was reversed, and the DNA was purified using a Monarch^®^ PCR & DNA Cleanup Kit (T1030S, NEB). The purified DNA products were subjected to PCR analysis using SYBR Green system (B21203, Biotool). Primers are listed in Table S3.

### Tissue microarray

The human NPC tissue microarray containing 110 samples of different clinical stages was obtained from Queen Mary Hospital, HKU. The staining was scored using a semiquantitative scoring system incorporating the proportion of the intensity of staining (0, no staining; 1, weak staining; 2, moderate staining; and 3, strong staining). The expression levels of the proteins were evaluated by multiplying the score of percentage of positive cell with the score of staining intensity and was evaluated in a blinded fashion (*H*-score = 1 × percentage of weak staining cell + 2 × percentage of moderate staining cell + 3 × percentage of strong staining cell). The protocol of immunohistochemical staining was performed as described previously [10, 42]. The use of clinical samples was approved by the Research Ethics Committee of HKU.

### Experimental in vivo metastasis study

Male nude or NOD/SCID mice at 6 weeks of age were housed in a specific pathogen-free room with the Guide for the Care and Use of Laboratory Animals of the University of Hong Kong. The mice were randomly divided into different groups before the injection. Luciferase-expressing C666-1 cells with different engineering constructs were resuspended in 100 µl PBS (1 × 10^6^ cells), and were then injected into the tail veins of mice. The mice were observed under the PE IVIS Spectrum in vivo imaging system (PerkinElmer) 6 weeks after injection. Ten weeks later, the mice were sacrificed, and the lungs were removed and fixed for hematoxylin and eosin staining.

### Statistical analysis

Data are expressed as means ± SD or SEM from three independent triplicates and were analyzed with GraphPad Prism 5 statistical software. The *χ*^2^-test was used to analyze the correlation between pPDHE1α-Ser293 and Snail expression and clinicopathological features. The overall survival probability of NPC patients was calculated using the Kaplan-Meier method and differences were compared using the log-rank test. The differences between experimental groups were analyzed with the Dennett *t* test; *p* values of 0.05 were considered statistically significant.

The details for western blot, RT-PCR, lentiviral package, luciferase report assay, wound healing, and migration/invasion assays are described in the Supplementary information.

## Supplementary information

Supplementary Information.

Supplementary Figure 1.

Supplementary Figure 2.

Supplementary Figure 3.

Supplementary Figure 4.

Supplementary Figure 5.

Supplementary Figure 6.

Supplementary Figure 7.

Supplementary Figure 8.

Supplementary Figure 9.

Supplementary tables.

Movie S1

Movie S2

Movie S3

Movie S4

Movie S5

Movie S6

Movie S7

Movie S8

Movie S9

## Data Availability

RNA sequencing raw data have been deposited at SRA with reference number as PRJNA515597.
