# Supplementary material for: mTORC2-mediated PDHE1α nuclear translocation links EBV-LMP1 reprogrammed glucose metabolism to cancer metastasis in nasopharyngeal carcinoma
Source: Oncogene. 2019 Feb 11;38(24):4669–84. doi: 10.1038/s41388-019-0749-y (PMC6756087; doi:10.1038/s41388-019-0749-y)
Supplement: Supplementary file 1 — Supplementary Information. [file 41388_2019_749_MOESM1_ESM.docx]

**Supplementary Information**

**Supplementary Materials and Methods**

**Lentivirus preparation**

Lentivirus preparation was performed as described previously [[1](#_ENREF_1)]. Briefly, the target gene constructs and the lentivirus packaging vectors, psPA2 and pMD2G, were transfected into the 293T packaging cell line using the X-treme GENE HP DNA transfection reagent (Roche, Basel, Switzerland) according to the manufacturer’s instructions. Three days after transfection, the supernatant was collected, filtered through a 0.45-μm filter, and used for infecting cells.

**Proliferation assay**

Cells were plated in a 96-well plate and used for experiments at 60% cell confluence. After treating the cells as indicated times, 20 μl of MTT (5 mg/ml) was added followed by 4 h incubation at 37°C. Then medium was removed and 150 μl DMSO was added to each well. Absorbance at 490 nm was recorded using a Micro-plate reader system (Victor^3^, PerkinElmer). The percentage of viable cells was calculated as follows: cell viability (%) = OD treatment / OD control × 100%.

**RT-PCR screening**

Cells at 80% confluence were harvested and total RNA was extracted by TRIzol (Roche). The extracted RNA was quantified and converted into cDNA. Quantitative real-time PCR of all samples was performed using a LightCycler 480 Probe Master Kit with the human universal probe library set (Roche Applied Science, Switzerland). Specific forward and reverse primers were designed with the assistance of ProbeFinder software (Roche Applied Science). The primer sequences used in qRT-PCR screening for this study are listed in **Table S3**.

**Western blotting**

After the indicated treatments, cells were harvested, washed with phosphate-buffered saline (PBS), and lysed in ice-cold radioimmunoprecipitation assay (RIPA) lysis buffer for 30 min. After protein quantification, 20 μg of denatured protein samples were subjected to SDS-PAGE. Western blot analysis was performed as described previously [[1](#_ENREF_1), [2](#_ENREF_2)]. The antibodies used in this study are listed in Table S2.

**Cloning of the constructs and mutant generation**

PCR amplification of the coding sequence region of PDHE1α gene was performed with Q5^®^ Hot Start High-Fidelity DNA Polymerase (M0493S, NEB). The PCR-amplified products were sub-cloned into the pcDNA3-3HA vector or pCDH-CMV-MCS-EF1 backbones between KpnI (R0142S, NEB) and XhoI (R0146S, NEB) using T4 DNA ligase (M0202S, NEB). Sanger DNA sequencing was used to verify the gene sequence. For mutant constructs, point mutations of PDHE1α at the serine293 residue to A or D were generated by PCR using the site mutation primers which are listed in Table S3. Other plasmids used in this study including shRictor #1 and #2 (#1384 and 1385), and pGL2-snail (#31694) were obtained from Addgene.

**Transwell migration, invasion and scrape-wound healing assays**

The Boyden chamber migration and invasion assays were performed using an 8.0 μm pore size transwell chamber (MCEP24H48, Millipore) with (for invasion assay) or without Matrigel coating (for migration assay). Each transwell chamber was inoculated with about 5,000 cells in 200 μl medium, and then inserted into a 24-well plate. After 48 h incubation, the trans-migrated cells to the lower surface were fixed in methanol and stained with crystal violet. The number of cells was counted in 3 random fields and representative images were captured. For scrape wound healing assay, confluent cells grown on 6-well plates were scraped with a yellow pipette tip. The closure of the scraped wound was monitored at indicated time points under a phase-contrast microscope.

**Luciferase reporter assay of *Snail* promoter**

The luciferase reporter assay was performed as previously described [[1](#_ENREF_1)]. The cells were seeded into a 12-well plate. The Promoter Reporter plasmids pGL2-snail (500 ng/well) and Renilla (100 ng/well) as an internal control were co-transfected into the 293T cells. The cells were treated for 36 hours. After treatment, luciferase activity was detected with a dual luciferase reporter assay system (E1910, Promega) following the manufacturer’s instructions.

**Measurements of glucose consumption and lactate production**

The glucose uptake and lactate production were measured as described previously [[1](#_ENREF_1)]. Briefly, Cells (5×10^5^) were seeded in each well of a 6-well plate and treated as indicated. After treatment, the medium was discarded and replaced with fresh medium, and cells were incubated for another 8 h. The culture medium was then collected for determination of glucose consumption and lactate levels using commercial assay kits for glucose and lactate (1200032002 for glucose uptake and 1200012002 for lactate production, Eton Bioscience, San Diego, CA). Glucose consumption and lactate production in the supernatant was calculated as the difference in incubated medium and fresh medium. The relative ratio of glucose and lactate was normalized by protein concentration of samples to exclude the impact of cell growth.

**Immunofluorescence staining**

For PDHE1α localization assay, cells grown on coverslips were pre-stained with MitoTracker Red CMXRos (Cell signaling, # 9082S) for 20 min in 37°C incubator, cells were then fixed with cold methanol for 10 min, permeabilized with 0.1% Triton X-100 for 10 min and then blocked with 3% BSA for 1 h at room temperature. Coverslips were incubated with primary antibodies against PDHE1α in room temperature for 3 h, followed by fluorescence dye-conjugated secondary antibody for 1 h in the dark. For phalloidin and gelatin digestion co-staining assay, cells were grown on FITC-conjugated gelatin coated coverslips for 24 h, then fixed with 4% formaldehyde for 10 min, followed by incubation with Fluo555-Phalloidin antibody for 1 h. The fluorescence images were acquired by confocal microscope (LSM 800, Zeiss, Germany). The nuclear intensity of the PDHE1α and the gelatin digestion area were quantitated by Image J.

**Supplementary Figure Legends**

**Supplementary Figure 1, Validation of EBV latent infection in immortalized NPE cells.** The EBV-infected NP361hTert, NP460hTert and NP550hTert cells were seeded in the 6-well plate. After attachment, (**A**) the brightfield and green channel (GFP) images were captured under a fluorescence microscope. The bottom panel shows the images of EBV FISH revealing the presence of EBV in the EBV-infected cells as punctate red dots. (**B**) The cells were lysed, and the cell lysate were subjected to western blot for analyzing the latent proteins with the specific antibodies. β-actin expression was used as the loading control. (**C**) The total RNA was extracted and EBV latent gene expressions in different cells were analyzed by qPCR. C666-1 was used as a positive control which contains endogenous EBV.

**Supplementary Figure 2,** **EBV infection induces glycolytic addiction in NPE cells.** Heatmap with full gene names and detailed description of genes involved in (**A**) glycolysis and (**B**) pyruvate metabolism signatures identified by GSEA analysis in three pairs of EBV negative and EBV positive cells. (**C**) qRT-PCR analysis of glycolytic and OXPHOS genes in NP460 cells infected with EBV and the control uninfected cell. (**D, H and K**) EBV-infected and uninfected NP460 cells, shLMP1-infected NP460-EBV cells, and LMP1-overexpressing NP69 cells were seeded, the ECAR and OCR were detected after the cells were treated with 2-DG and Oligomycin for 24 h. (**E** and **I**) NP361/NP361-EBV & NP550/ NP550-EBV cells, and the EBV-infected NPE cells with or without LMP1 knockdown were plated at 10,000 cells/well for 24 h, then the cells were incubated with the ECAR or OCR reagents and the values were measured simultaneously by using the 96 well plate reading system (Victor, PerkinElmer) in real time. (**F**) Three pairs of EBV-infected NPE cells as well as the C666-1 cells were infected with shLMP1 and control empty retroviral vector, the cellular RNA was extracted, and the RNA was subjected to qPCR analysis for LMP1 expression. (**G**) RT-PCR analysis of the metabolic genes in NP460 and NP460-EBV cells with or without LMP1 knockdown. (**J**) NP460 and NP69 cells with stable overexpression of LMP1 were harvested for RT-PCR analysis of metabolism-associated genes expression. Data are means ± SD. *, *p*<0.05; **, *p*<0.01; ***, *p*<0.005.

**Supplementary Figure 3,** **LMP1 upregulates glycolysis in NPE cells enhances cell motility.** (**A**) Control and LMP1-expressing cells were assessed for migration ability by wound healing assay. Cells were treated with 2-DG or STF-31 to block the glucose metabolic process and the confluent monolayer was scraped with a sterile yellow tip. The cell migration to the scraped area was measured at the indicated time points.

**Supplementary Figure 4,** **mTORC2 activation by LMP1 involves in metabolism and cell motility.** Heatmap with full gene names and detailed description of genes involved in (**A**) PI3K-AKT-mTOR and (**B**) mTOR signaling signatures identified by GSEA analysis in three pairs of EBV-negative and EBV-positive cells. (**C**) Migration and invasion of NP69-LMP1 cells were assayed after transfection of different shRNAs as well as addition of rapamycin to block the mTORC1 and mTORC2 signaling activation. The number of cells in five random microscopic fields were counted for each group. (**D** and **E**) NP69-LMP1 cells were transfected with shRictor and the confluent monolayer cells were scraped using a yellow tip. The cells migration into the wounded monolayer was assessed at 24 and 48 hours after scraping, and the wound closure areas were statistical analyzed. Data are means ± SD. *, *p*<0.05; **, *p*<0.01; ***, *p*<0.005.

**Supplementary Figure 5,** **AKT activation mediates glucose metabolism and cell motility.** NP69 cells were transfected with AKT constructs or treated with AKT inhibitor for 48 h, and then subjected to following experiments: (**A** and **B**) cells were lysed and the lysates were subjected to western blot to analyze the indicated targets using specific antibodies; (**C**) the glucose uptake and lactate production were analyzed; (**D**) the cell migration, invasion and wound healing were monitored with reprehensive images taken; (**E**) the migrated and invasive cells were counted and (**F**) the wound recovery rate was quantified; (**G**) Representative tracks of cell movements that were traced and visualized using metaphase software every 10 min for 24 h. The accumulated distance was analyzed by metaphase software. Data are means ± SD (means ± SEM for Figure 5G). *, *p*<0.05; **, *p*<0.01; ***, *p*<0.005.

**Supplementary Figure 6, LMP1 activates mTORC2/AKT signaling by autocrine secretion of IGF1.** (**A**) Experimental design showing the protocol to harvest different conditioned medium for functional assay. (**B**) Different pairs of NPE and NPC cells were cultured for 48 h, and then their culture medium were harvested for ELISA assay using IGF-1 specific commercial kit. (**C**) NP69-pLNSX and LMP1 cells were treated with different doses of AG-1024 and antibodies against-IgG or IGF-1 for indicated time points, followed by detection of viability using MTT assay; (**D**) the culture media were harvested for measurement of the glucose uptake and lactate production using commercial kits; (**E** and **F**) the cells were lysed and analyzed by specific antibodies against glycolysis and cell motility; (**G**) cell migration and invasion were assayed using Transwell chambers without or with coated Matrigel respectively. Representative images of the bottom surface are shown. The number of cells in five random microscopic fields were counted for each group. Data are means ± SD. *, *p*<0.05; **, *p*<0.01; ***, *p*<0.005.

**Supplementary Figure 7,** **Nuclear translocation of PDHE1α mediated by mTORC2/AKT signaling is involved in mediating LMP1-enhanced motility in NPE cells.** (**A**) Cells were treated with shRictor lentivirus or (**B**) transfected with PDHK1 constructs for 48 h. Cells were then harvested and isolated into different fractions for western blot analysis of the indicated targets.

**Supplementary Figure 8,** **Nuclear PDHE1α promotes histone acetylation of *Snail* promoter to mediate LMP1-enhanced cell motility.** (**A**) NP69 cells treated with AKT inhibitors or transfected with AKT constructs were analyzed by western blot using the indicated antibodies. NP69 cells transfected with LMP1 were then infected with (**B**) shRictor or (**C**) PDHK1-associated constructs, followed by western blot analysis using the indicated antibodies. (**D**) After transfecting the 293T cells with LMP1 and pGL2-Snail, they were then treated with AKT inhibitors or constructs (left panel), infected with lentivirus against Rictor (middle panel) as well as PDHK1-associated constructs (right panel). After treatment, cells were lysed, and the luciferase activity was measured using a commercial kit. Data are means ± SD. *, *p*<0.05; **, *p*<0.01; ***, *p*<0.005.

**Supplementary Figure 9,** **The correlation between LMP1 expression and IGF1-AKT-PDHE1α-Snail axis.** LMP1 expression was positively correlated with IGF1 (p=0.0038) and pAKT Ser473 (p=0.0085) but not pPDHE1α Ser293 (p=0.1069) and Snail (p=0.0523).

**Supplementary Movies 1-9,**

**Movie S1 to 4**, NP69-LNSX cell (Movie 1) and NP69-LMP1 cells (Movie 2-4) were seeded on the coverglass chamber. After attachment, cells were incubated with 2-DG (Movie 3) and STF-31 (Movie 4), and were observed under a time-lapse microscope. Representative tracks of cell movements were traced and visualized using metaphase software every 10 min for 24 h.

**Movie S5 to 9**, NP460-LPCX (Movie 5), NP460-LMP1 (Movie 6), NP460-LMP1-shScr (Movie 7), NP460-LMP1-shRic#1 (Movie 8) and NP460-LMP1-shRic#2 (Movie 9) were seeded on the coverglass chamber and were observed under time-lapse microscope. Representative tracks of cell movements were traced and visualized using metaphase software every 10 min for 24 h.

**Reference**

1. Zhang, J., L. Jia, W. Lin, Y.L. Yip, K.W. Lo, V.M. Lau, et al., Epstein-Barr Virus-Encoded Latent Membrane Protein 1 Upregulates Glucose Transporter 1 Transcription via the mTORC1/NF-kappaB Signaling Pathways. J Virol 2017; 91.

2. Zhu, D.D., J. Zhang, W. Deng, Y.L. Yip, H.L. Lung, C.M. Tsang, et al., Significance of NF-kappaB activation in immortalization of nasopharyngeal epithelial cells. Int J Cancer 2016; 138: 1175-85.
