# Supplementary figures and images for: mTORC2-mediated PDHE1α nuclear translocation links EBV-LMP1 reprogrammed glucose metabolism to cancer metastasis in nasopharyngeal carcinoma
Source: Oncogene. 2019 Feb 11;38(24):4669–84. doi: 10.1038/s41388-019-0749-y (PMC6756087; doi:10.1038/s41388-019-0749-y)

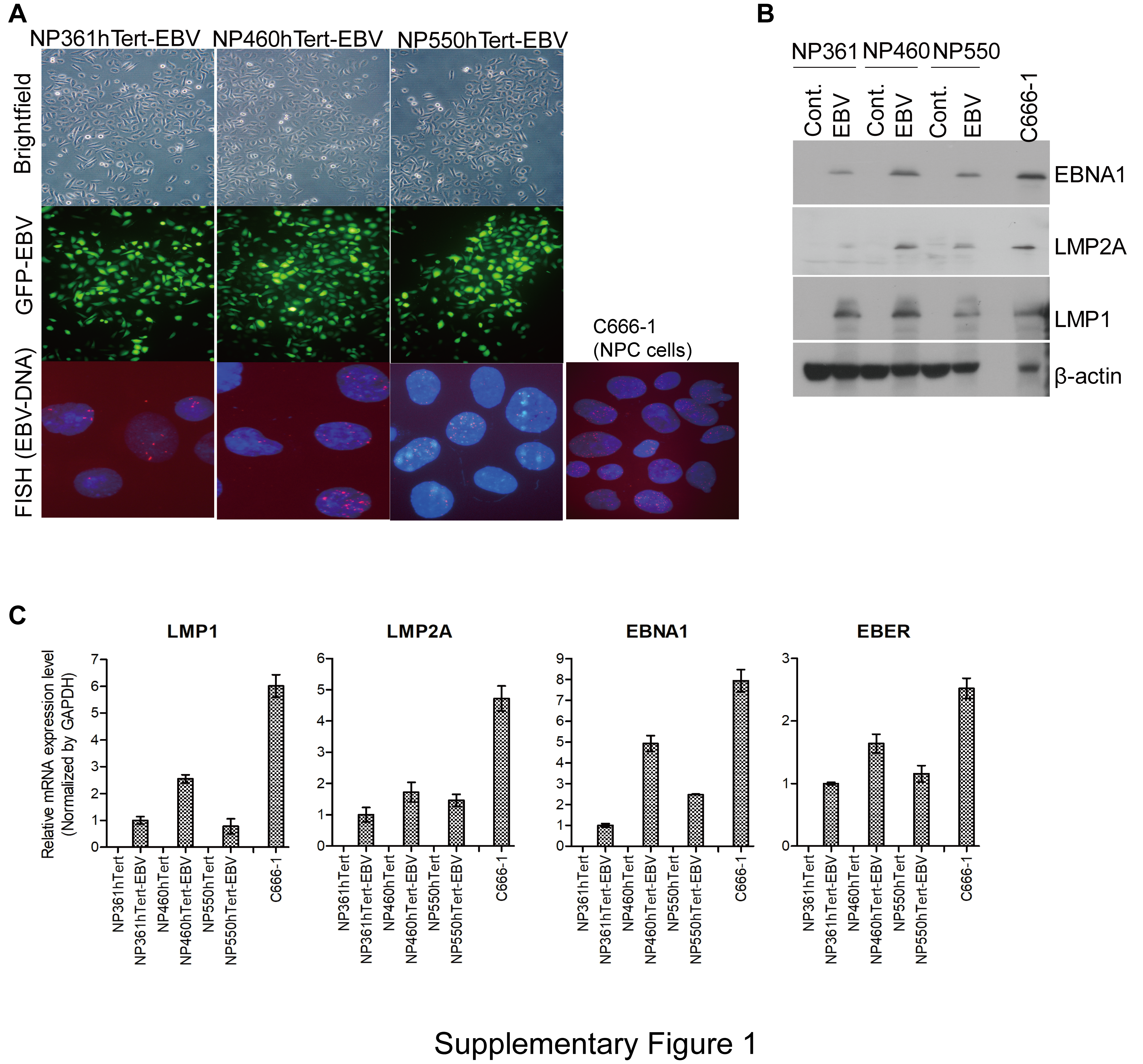

Supplement: Supplementary file 2 — Supplementary Figure 1. [file 41388_2019_749_MOESM2_ESM.tif]

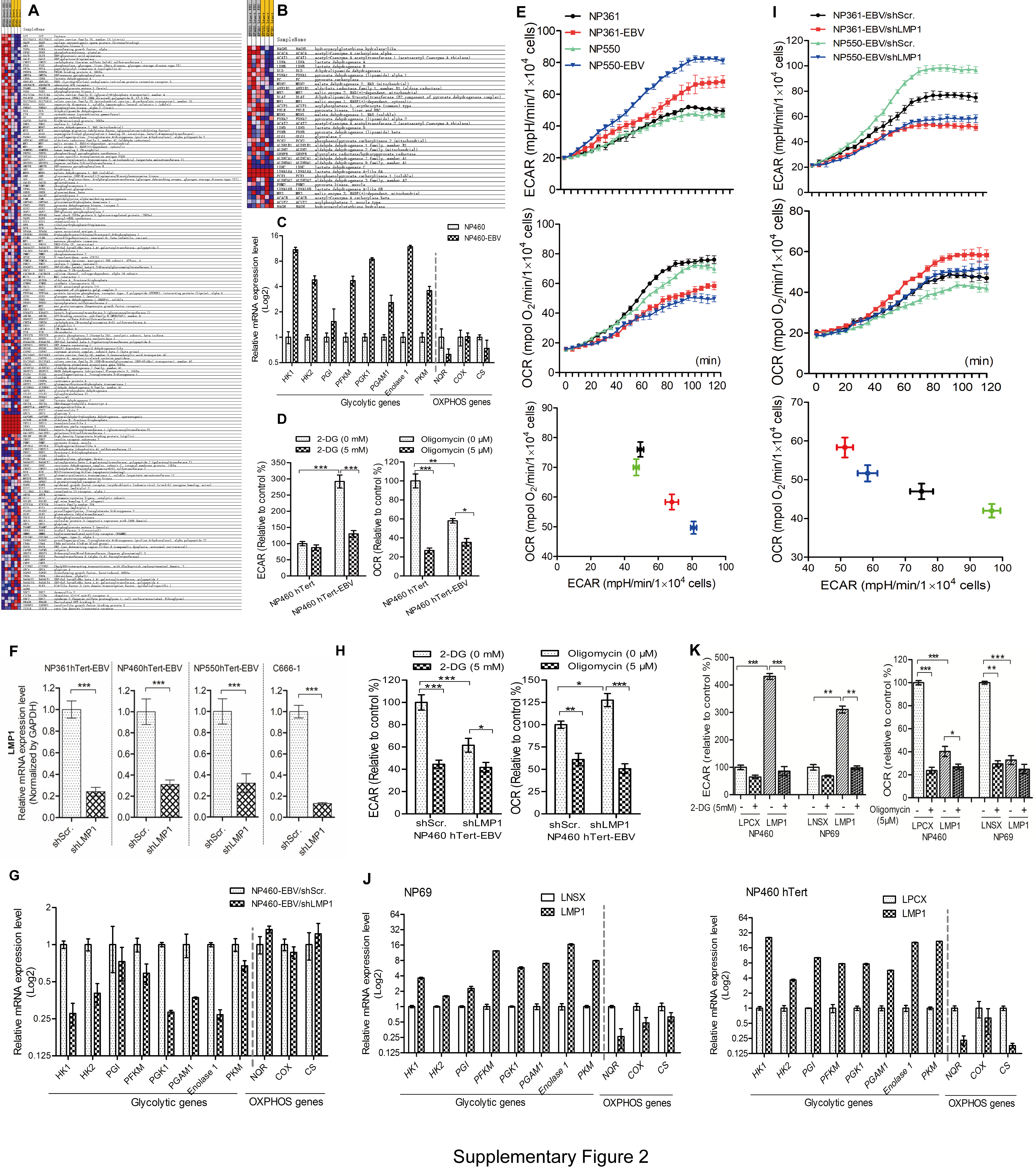

Supplement: Supplementary file 3 — Supplementary Figure 2. [file 41388_2019_749_MOESM3_ESM.tif]

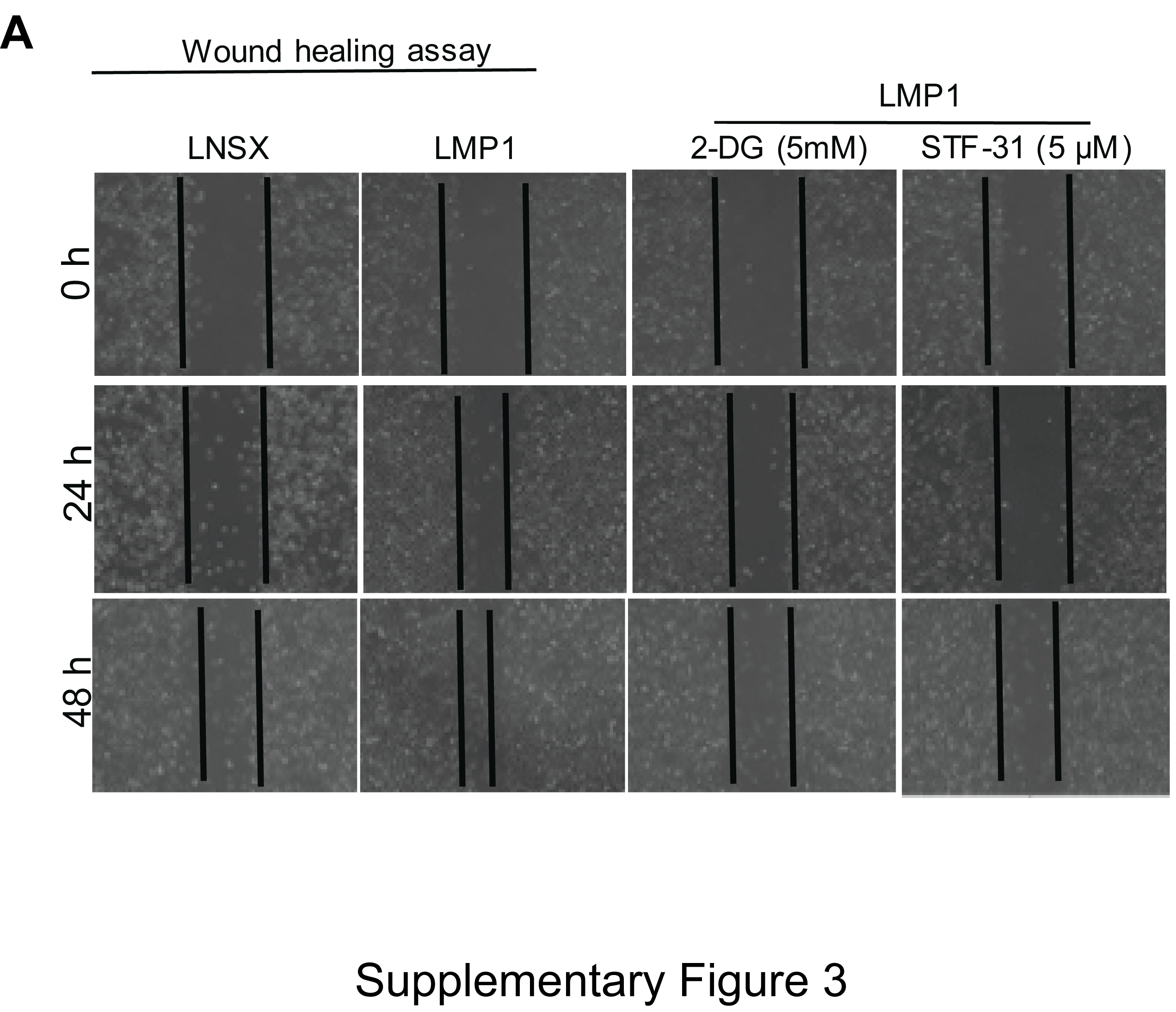

Supplement: Supplementary file 4 — Supplementary Figure 3. [file 41388_2019_749_MOESM4_ESM.tif]

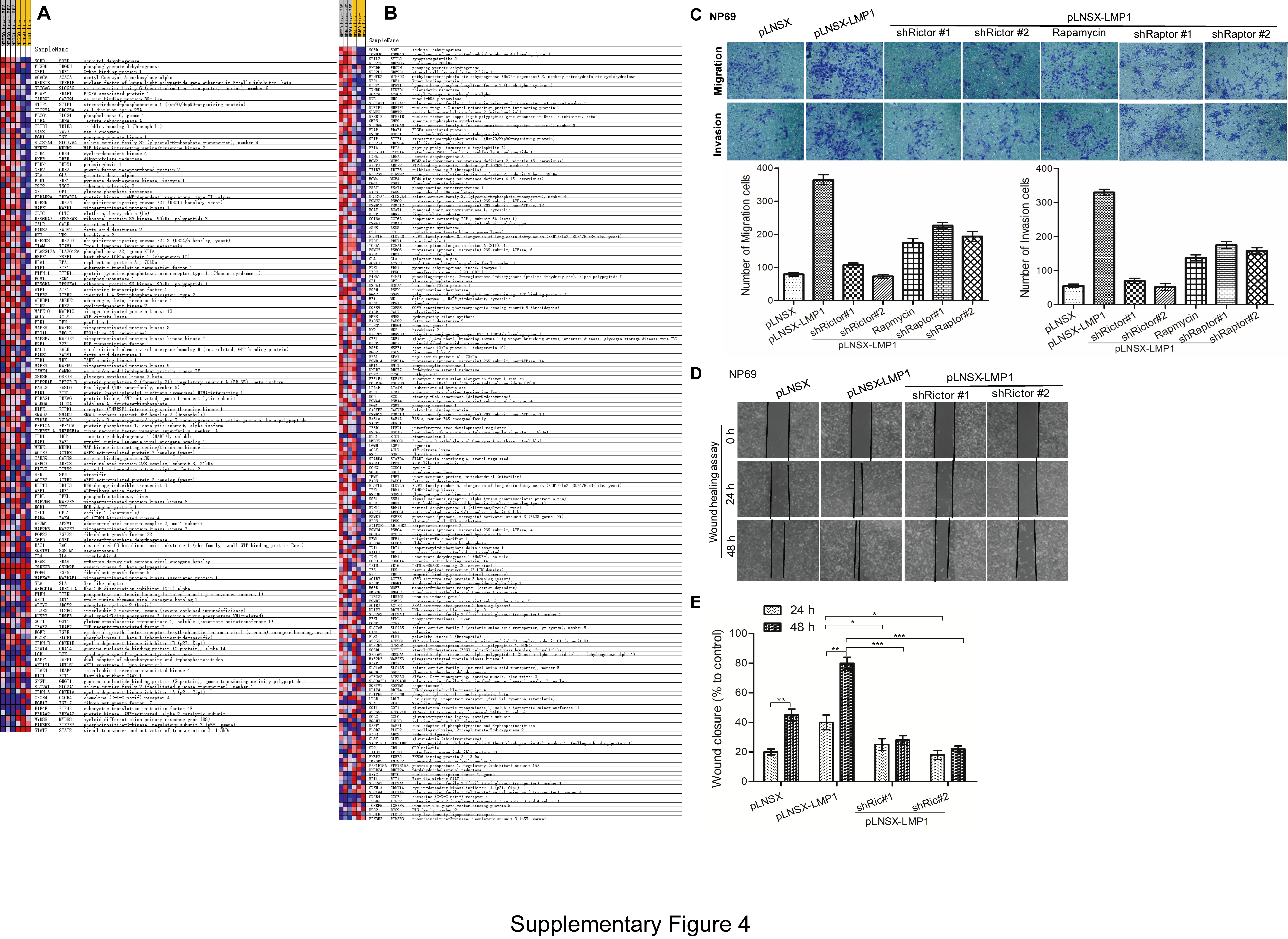

Supplement: Supplementary file 5 — Supplementary Figure 4. [file 41388_2019_749_MOESM5_ESM.tif]

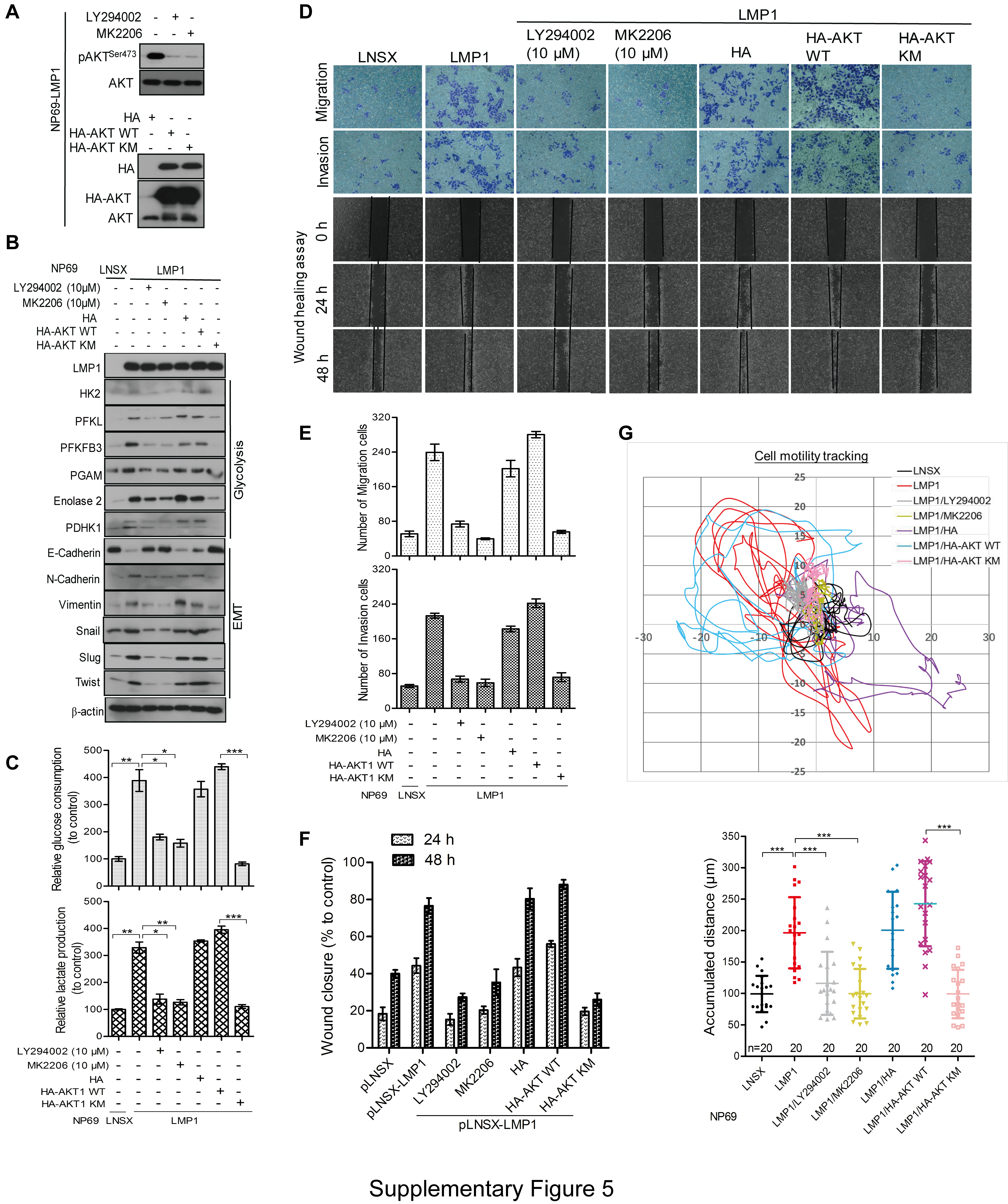

Supplement: Supplementary file 6 — Supplementary Figure 5. [file 41388_2019_749_MOESM6_ESM.tif]

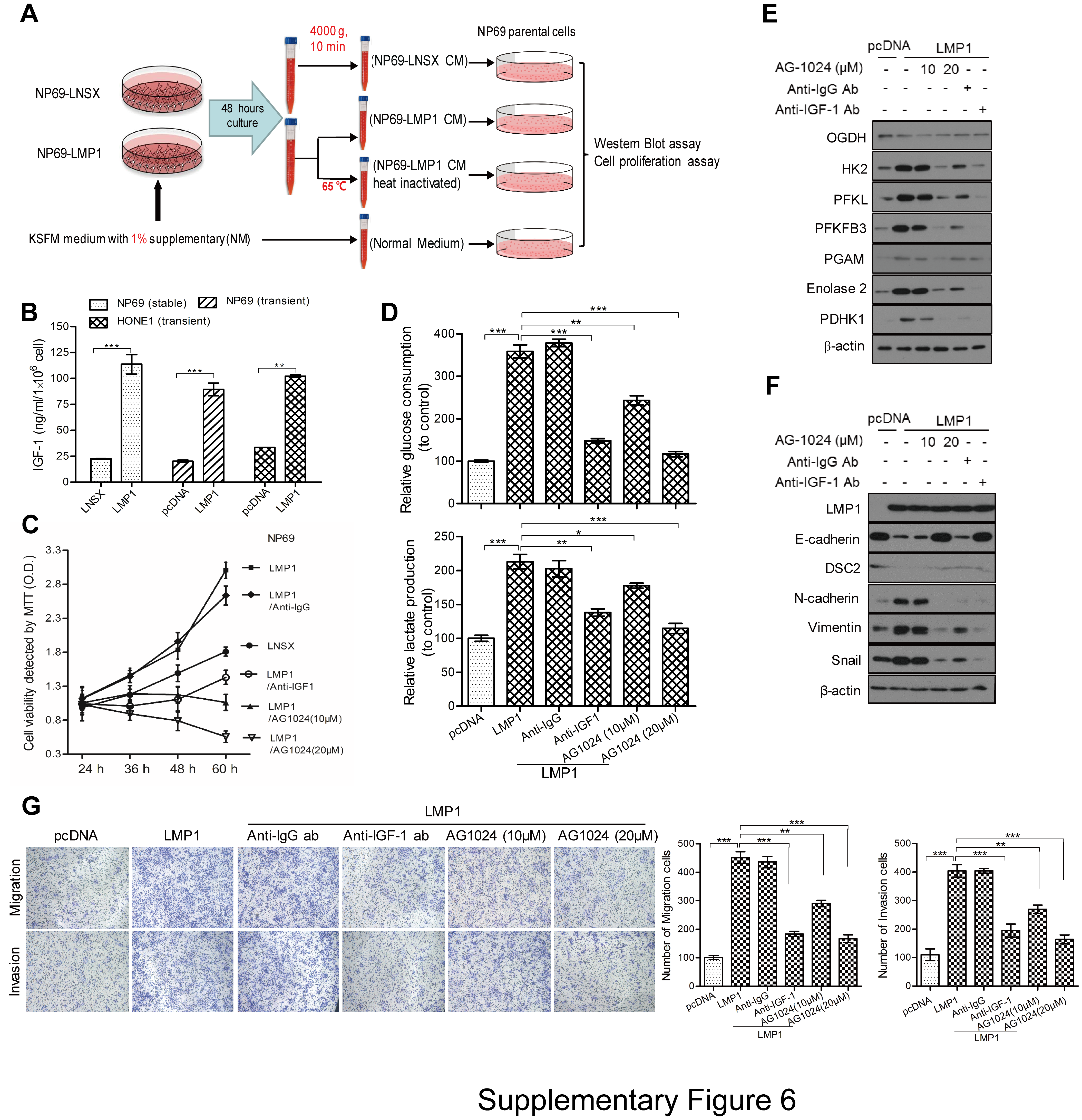

Supplement: Supplementary file 7 — Supplementary Figure 6. [file 41388_2019_749_MOESM7_ESM.tif]

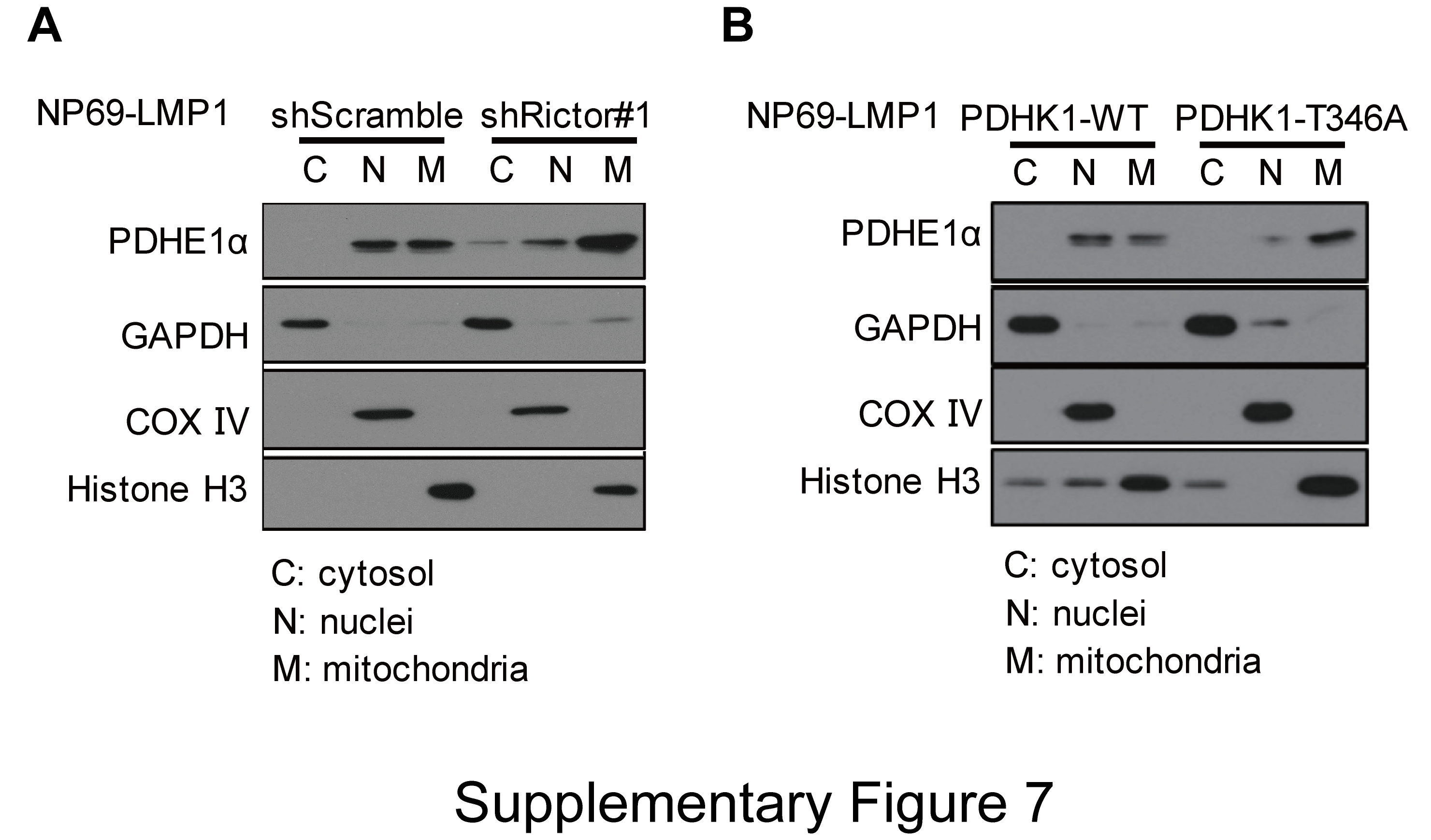

Supplement: Supplementary file 8 — Supplementary Figure 7. [file 41388_2019_749_MOESM8_ESM.tif]

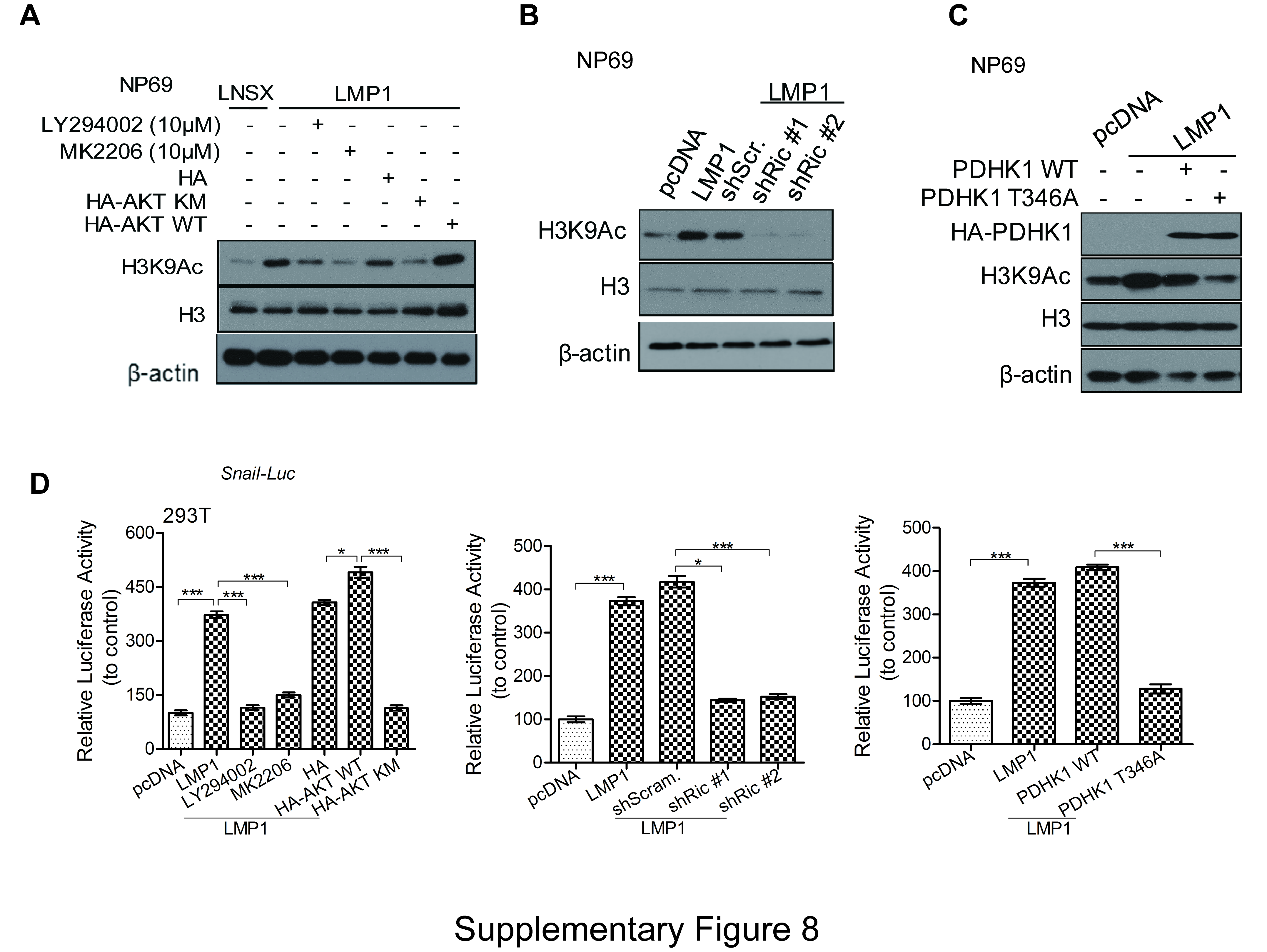

Supplement: Supplementary file 9 — Supplementary Figure 8. [file 41388_2019_749_MOESM9_ESM.tif]

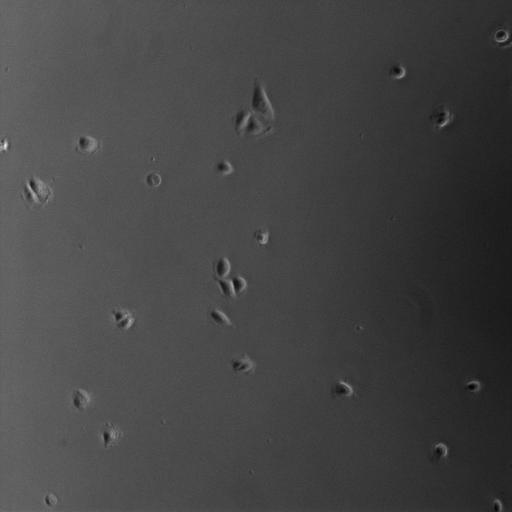

Supplement: Supplementary file 12 — Movie S1 [file 41388_2019_749_MOESM12_ESM.gif]

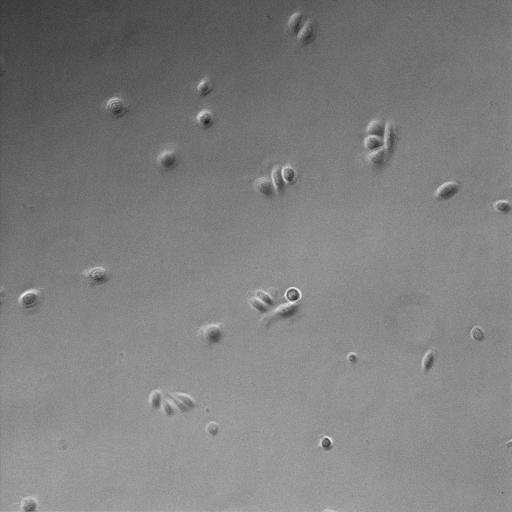

Supplement: Supplementary file 13 — Movie S2 [file 41388_2019_749_MOESM13_ESM.gif]

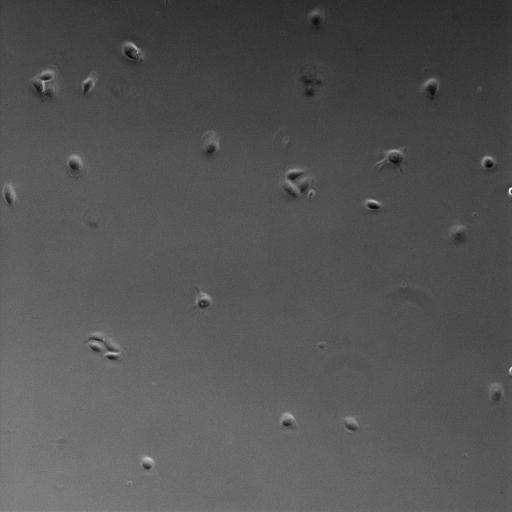

Supplement: Supplementary file 14 — Movie S3 [file 41388_2019_749_MOESM14_ESM.gif]

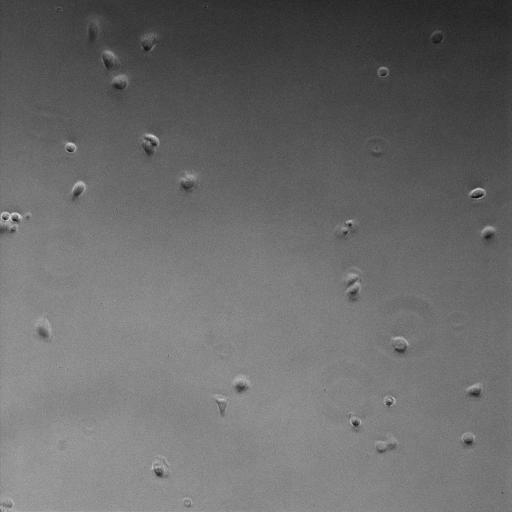

Supplement: Supplementary file 15 — Movie S4 [file 41388_2019_749_MOESM15_ESM.gif]

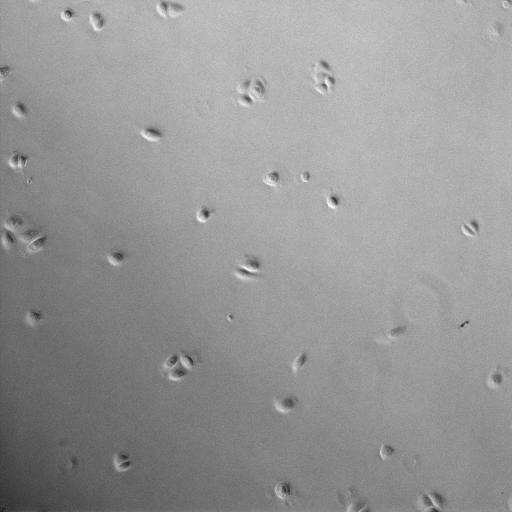

Supplement: Supplementary file 16 — Movie S5 [file 41388_2019_749_MOESM16_ESM.gif]

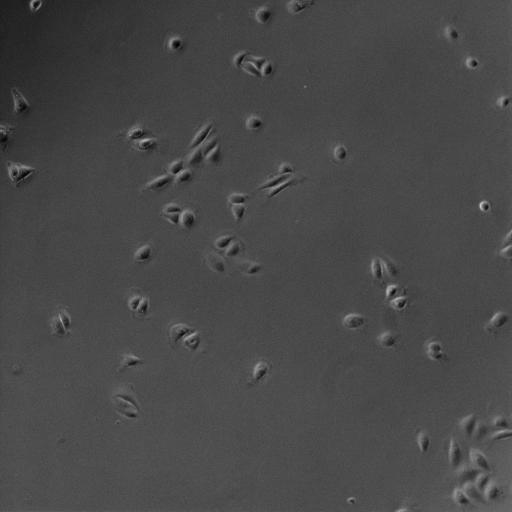

Supplement: Supplementary file 17 — Movie S6 [file 41388_2019_749_MOESM17_ESM.gif]

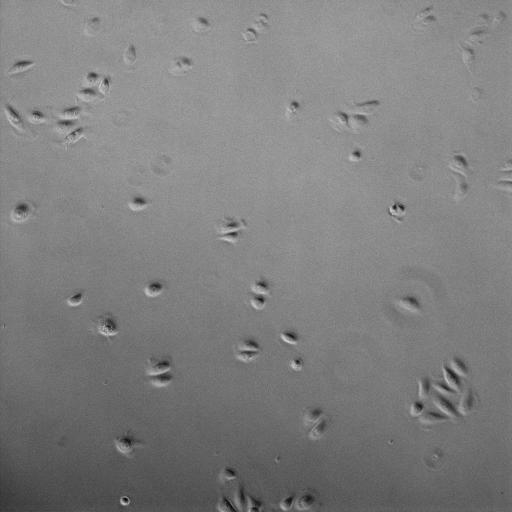

Supplement: Supplementary file 18 — Movie S7 [file 41388_2019_749_MOESM18_ESM.gif]

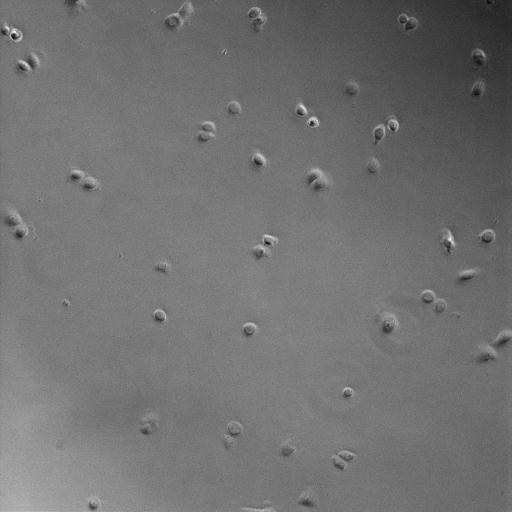

Supplement: Supplementary file 19 — Movie S8 [file 41388_2019_749_MOESM19_ESM.gif]

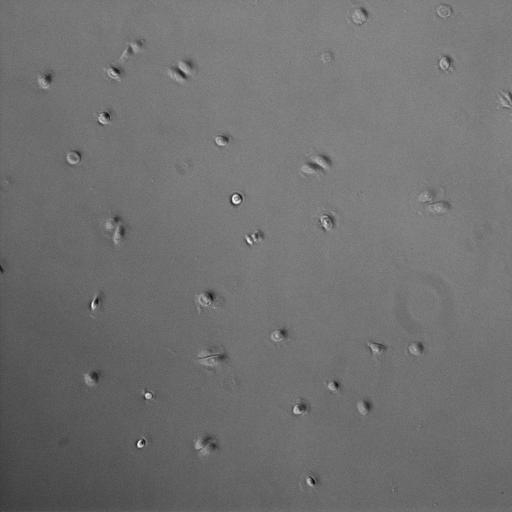

Supplement: Supplementary file 20 — Movie S9 [file 41388_2019_749_MOESM20_ESM.gif]
