# Supplementary material for: mTORC2-mediated PDHE1α nuclear translocation links EBV-LMP1 reprogrammed glucose metabolism to cancer metastasis in nasopharyngeal carcinoma
Source: Oncogene. 2019 Feb 11;38(24):4669–84. doi: 10.1038/s41388-019-0749-y (PMC6756087; doi:10.1038/s41388-019-0749-y)
Supplement: Supplementary file 11 — Supplementary tables. [file 41388_2019_749_MOESM11_ESM.docx]

| **Table S1, Gene lists**  **Genes downregulated** | | |
| --- | --- | --- |
| **transcript_ID** | **gene** | **Description** |
| NM_015430 | ADAR | peptidase domain containing associated with muscle regeneration 1 |
| NM_001184900 | CNOT4 | caspase recruitment domain family, member 8 |
| NM_197955 | CARKD | chromosome 15 open reading frame 48 |
| NM_001017999 | LOC100286793 | kazrin, periplakin interacting protein |
| NM_001166624 | CYP2C8 | complement factor H-related 3 |
| NM_001199172 | NPDC1 | Mannosyl (alpha-1,6-)-glycoprotein beta-1,6-N-acetyl-glucosaminyltransferase, isozyme B |
| NM_001206609 | SMPD1 | selectin P ligand |
| NM_001198943 | FAM66E | dystrobrevin, alpha |
| NM_001199777 | RIPK4 | POC1 centriolar protein homolog B (Chlamydomonas) |
| NM_000673 | ATP6V1E1 | alcohol dehydrogenase 7 (class IV), mu or sigma polypeptide |
| NM_172206 | CLIP2 | calcium/calmodulin-dependent protein kinase kinase 1, alpha |
| NM_001145672 | TIMP3 | sorbin and SH3 domain containing 2 |
| NM_001128177 | ANKRD13A | thyroid hormone receptor, beta (erythroblastic leukemia viral (v-erb-a) oncogene homolog 2, avian) |
| NM_006091 | DPP3 | coronin, actin binding protein, 2B |
| NM_201544 | MAF | lectin, galactoside-binding, soluble, 8 |
| NM_015925 | MYCL1 | lipolysis stimulated lipoprotein receptor |
| NM_001194992 | NKX1-2 | methylphosphate capping enzyme |
| NM_001167987 | LEPRE1 | inhibitor of CDK, cyclin A1 interacting protein 1 |
| NM_014659 | RNLS | diphosphoinositol pentakisphosphate kinase 1 |
| NM_001177381 | KIAA0913 | cytoplasmic polyadenylation element binding protein 2 |
| NM_001135936 | RNF146 | periostin, osteoblast specific factor |
| NR_029393 | LOC728192 | keratin 16 pseudogene 3 |
| NM_001111309 | PRR15 | phosphodiesterase 4A, cAMP-specific |
| NM_004735 | MXD4 | leucine rich repeat (in FLII) interacting protein 1 |
| NM_020929 | MUM1 | leucine rich repeat containing 4C |
| NM_000216 | LOC100130899 | Kallmann syndrome 1 sequence |
| NM_018689 | LOC145474 | KIAA1199 |
| NM_006329 | GGA1 | fibulin 5 |
| NM_001113561 | SGPP2 | ring finger protein 180 |
| NM_004613 | TSC22D1 | transglutaminase 2 (C polypeptide, protein-glutamine-gamma-glutamyltransferase) |
| NM_001001991 | ADAMTSL3 | peptidase domain containing associated with muscle regeneration 1 |
| NM_058229 | GLTSCR1 | F-box protein 32 |
| NM_003020 | SLC47A2 | secretogranin V (7B2 protein) |
| NM_001085 | SNORA42 | serpin peptidase inhibitor, clade A (alpha-1 antiproteinase, antitrypsin), member 3 |
| NR_027706 | GPX4 | hypothetical FLJ11235 |
| NM_001127598 | LACTB | insulin-like growth factor 2 (somatomedin A) |
| NM_005764 | PTPRJ | PDZK1 interacting protein 1 |
| NM_024764 | COL21A1 | cation channel, sperm-associated, beta |
| NM_000132 | FRZB | coagulation factor VIII, procoagulant component |
| NM_030583 | ABHD8 | matrilin 2 |
| NM_033378 | CYP2J2 | chorionic gonadotropin, beta polypeptide 2 |
| NM_198541 | LAMB3 | IGF-like family member 1 |
| NM_022147 | SLC11A1 | receptor (chemosensory) transporter protein 4 |
| NM_144594 | IQGAP2 | gametocyte specific factor 1 |
| NM_199511 | CRABP2 | coiled-coil domain containing 80 |
| NM_001146255 | RNASEK | podocan-like 1 |
| NM_198721 | DNAJB5 | collagen, type XXV, alpha 1 |
| NM_002964 | SLC22A23 | S100 calcium binding protein A8 |
| NM_004840 | C15orf37 | Rac/Cdc42 guanine nucleotide exchange factor (GEF) 6 |
| NM_002272 | LOC728752 | keratin 4 |
| NM_030915 | LYSMD1 | limb bud and heart development homolog (mouse) |
| NM_001135599 | ANGPT1 | transforming growth factor, beta 2 |
| NR_003542 | TAPBPL | proteoglycan 3 pseudogene |
| NM_014622 | ZNF812 | von Willebrand factor A domain containing 5A |
| NM_002959 | TLN1 | sortilin 1 |
| NR_026586 | FBLN7 | EF-hand calcium binding domain 2 |
| NM_053026 | ACP2 | myosin light chain kinase |
| NM_001128176 | ANKRD1 | thyroid hormone receptor, beta (erythroblastic leukemia viral (v-erb-a) oncogene homolog 2, avian) |
| NM_004624 | ZNF671 | vasoactive intestinal peptide receptor 1 |
| NM_005621 | SLC22A18 | S100 calcium binding protein A12 |
| NM_080872 | ZNF438 | unc-5 homolog D (C. elegans) |
| NM_138450 | C15orf62 | ADP-ribosylation factor-like 11 |
| NM_022661 | TMEM104 | SPANX family, member C |
| NM_017418 | EXD2 | deleted in esophageal cancer 1 |
| NM_057166 | DNAJC4 | collagen, type VI, alpha 3 |
| NM_004360 | CXCL14 | cadherin 1, type 1, E-cadherin (epithelial) |
| NM_214461 | CD99L2 | chromosome 2 open reading frame 27B |
| NM_152456 | LBH | interleukin 34 |
| NM_018927 | PPP2R3B-AS1 | protocadherin gamma subfamily B, 7 |
| NM_002899 | SEC14L1 | retinol binding protein 1, cellular |
| NM_001080433 | CRIM1 | coiled-coil domain containing 85A |
| NM_001178102 | ABHD4 | lysyl oxidase |
| NM_002353 | TP53INP2 | tumor-associated calcium signal transducer 2 |
| NM_004588 | SLCO2A1 | sodium channel, voltage-gated, type II, beta |
| NM_000775 | ENSA | cytochrome P450, family 2, subfamily J, polypeptide 2 |
| NM_170693 | SNORD12 | serum/glucocorticoid regulated kinase 2 |
| NM_001206615 | FGR | ets homologous factor |
| NM_001490 | IFI27L2 | glucosaminyl (N-acetyl) transferase 1, core 2 |
| NM_004170 | STAT3 | solute carrier family 1 (neuronal/epithelial high affinity glutamate transporter, system Xag), member 1 |
| NM_182511 | COL25A1 | cerebellin 2 precursor |
| NM_001145635 | WDR91 | tripartite motif containing 45 |
| NM_001135775 | FAM89B | chromosome 9 open reading frame 7 |
| NM_001130088 | ABLIM2 | actin binding LIM protein family, member 2 |
| NM_130850 | C9orf169 | bone morphogenetic protein 4 |
| NM_000087 | DLGAP4 | cyclic nucleotide gated channel alpha 1 |
| NM_005651 | TRIM45 | tryptophan 2,3-dioxygenase |
| NR_038952 | MFGE8 | hypothetical LOC100506343 |
| NM_006226 | RFTN2 | phospholipase C-like 1 |
| NM_182762 | MYL6 | metastasis associated in colon cancer 1 |
| NM_001005289 | PLB1 | olfactory receptor, family 52, subfamily H, member 1 |
| NM_207517 | ATP6V0D1 | ADAMTS-like 3 |
| NM_003728 | ZNF418 | unc-5 homolog C (C. elegans) |
| NM_004822 | PHLDA3 | netrin 1 |
| NM_001935 | FAM65A | dipeptidyl-peptidase 4 |
| NM_002965 | SLC24A3 | S100 calcium binding protein A9 |
| NM_001160333 | PDGFC | neurofascin |
| NM_004932 | CXCL16 | cadherin 6, type 2, K-cadherin (fetal kidney) |
| NM_001004439 | LOC100093631 | integrin, alpha 11 |
| NM_001130986 | MYLK | dysferlin, limb girdle muscular dystrophy 2B (autosomal recessive) |
| NM_001100594 | THRA | SNF related kinase |
| NM_198545 | CCND3 | chromosome 1 open reading frame 187 |
| NM_003661 | C10orf68 | apolipoprotein L, 1 |
| NM_007168 | ARMC7 | ATP-binding cassette, sub-family A (ABC1), member 8 |
| NM_014862 | C16orf96 | aryl-hydrocarbon receptor nuclear translocator 2 |
| NM_005100 | BBC3 | A kinase (PRKA) anchor protein 12 |
| NM_001204376 | ADAMTS17 | natriuretic peptide receptor C/guanylate cyclase C (atrionatriuretic peptide receptor C) |
| NM_001081492 | ABCA5 | keratin 80 |
| NM_001146 | BRWD1 | angiopoietin 1 |
| NM_030968 | CRAT | C1q and tumor necrosis factor related protein 1 |
| NM_033260 | HENMT1 | forkhead box Q1 |
| NM_153000 | C10orf54 | adenomatosis polyposis coli down-regulated 1 |
| NM_033512 | ZC3H10 | TSPY-like 5 |
| NM_198493 | BTC | ankyrin repeat domain 45 |
| NR_024447 | MAU2 | hypothetical LOC100128288 |
| NM_013358 | PLXNA3 | peptidyl arginine deiminase, type I |
| NM_001193329 | CHP | chromosome 9 open reading frame 3 |
| NM_018401 | TMEM86A | serine/threonine kinase 32B |
| NM_001113207 | ZER1 | thiosulfate sulfurtransferase (rhodanese)-like domain containing 1 |
| NM_004120 | IDUA | guanylate binding protein 2, interferon-inducible |
| NM_014266 | ITGA3 | hematopoietic cell signal transducer |
| NR_033939 | MBD6 | hypothetical LOC100129858 |
| NR_024151 | KIAA1244 | heat shock 70kDa protein 7 (HSP70B) |
| NM_032488 | DKK3 | cornifelin |
| NM_001098520 | KIAA1522 | HIV-1 Tat interactive protein 2, 30kDa |
| NM_006633 | LINC00256A | IQ motif containing GTPase activating protein 2 |
| NM_002332 | MTHFS | low density lipoprotein receptor-related protein 1 |
| NM_020633 | ZNF740 | vomeronasal 1 receptor 1 |
| NM_001199758 | P4HA2 | 5,10-methenyltetrahydrofolate synthetase (5-formyltetrahydrofolate cyclo-ligase) |
| NM_005630 | TANC1 | solute carrier organic anion transporter family, member 2A1 |
| NR_038995 | MAP3K8 | long intergenic non-protein coding RNA 327 |
| NM_004112 | GPR132 | fibroblast growth factor 11 |
| NM_012244 | TAGLN | solute carrier family 7 (amino acid transporter light chain, L system), member 8 |
| NM_001017920 | EPHB1 | death associated protein-like 1 |
| NM_022036 | IL6 | G protein-coupled receptor, family C, group 5, member C |
| NM_153611 | EIF1B | cytochrome b, ascorbate dependent 3 |
| NM_182946 | PDZK1IP1 | ninein (GSK3B interacting protein) |
| NM_024600 | UBA1 | transmembrane protein 204 |
| NM_021992 | UCN2 | thymosin beta 15a |
| NM_020639 | SESN2 | receptor-interacting serine-threonine kinase 4 |
| NM_004693 | LOC80054 | keratin 75 |
| NM_000855 | IRF6 | guanylate cyclase 1, soluble, alpha 2 |
| NM_138445 | IGSF10 | G protein-coupled receptor 146 |
| NM_001848 | DNAJC28 | collagen, type VI, alpha 1 |
| NM_001080482 | CHMP2A | chromosome 9 open reading frame 172 |
| NM_001242883 | COL1A1 | carbohydrate kinase domain containing |
| NR_028287 | HS6ST1 | GABA(A) receptors associated protein like 3, pseudogene |
| NM_001204375 | ACVR1 | natriuretic peptide receptor C/guanylate cyclase C (atrionatriuretic peptide receptor C) |
| NM_000228 | LRRC8A | laminin, beta 3 |
| NM_001161778 | PTMS | pyruvate dehyrogenase phosphatase catalytic subunit 1 |
| NM_001130985 | MX1 | dysferlin, limb girdle muscular dystrophy 2B (autosomal recessive) |
| NM_032621 | C5orf62 | brain expressed X-linked 2 |
| NM_021202 | VAMP5 | tumor protein p53 inducible nuclear protein 2 |
| NR_027232 | RPL32P3 | PPP2R3B antisense RNA 1 (non-protein coding) |
| NM_004101 | FRMD8 | coagulation factor II (thrombin) receptor-like 2 |
| NM_015669 | PPIP5K1 | protocadherin beta 5 |
| NM_012418 | HINFP | fascin homolog 2, actin-bundling protein, retinal (Strongylocentrotus purpuratus) |
| NM_207334 | GALNT2 | family with sequence similarity 43, member B |
| NM_198593 | CPEB2 | C1q and tumor necrosis factor related protein 1 |
| NM_024690 | PAK6 | mucin 16, cell surface associated |
| NM_006308 | KIAA1310 | heat shock 27kDa protein 3 |
| NM_001005915 | FOXD4L5 | v-erb-b2 erythroblastic leukemia viral oncogene homolog 3 (avian) |
| NM_015065 | FPGS | exophilin 5 |
| NM_182507 | ABAT | keratin 80 |
| NM_001143839 | PROS1 | phosphodiesterase 2A, cGMP-stimulated |
| NM_014391 | BSDC1 | ankyrin repeat domain 1 (cardiac muscle) |
| NM_021244 | SIRT2 | Ras-related GTP binding D |
| NM_199262 | TMCC2 | Sp6 transcription factor |
| NM_001124 | ATXN2L | adrenomedullin |
| NM_002317 | ABCD1 | lysyl oxidase |
| NM_198332 | AMIGO1 | signal transducer and activator of transcription 2, 113kDa |
| NR_027424 | GBP2 | family with sequence similarity 66, member E |
| NM_203281 | C9orf172 | BMX non-receptor tyrosine kinase |
| NM_025041 | CDK18 | chromosome 3 open reading frame 36 |
| NM_138621 | C4orf49 | BCL2-like 11 (apoptosis facilitator) |
| NM_178031 | ANKRD45 | transmembrane protein 132A |
| NM_022059 | EHBP1 | chemokine (C-X-C motif) ligand 16 |
| NM_005461 | NAPA | v-maf musculoaponeurotic fibrosarcoma oncogene homolog B (avian) |
| NM_001242827 | SPPL2B | sirtuin 5 |
| NM_014858 | TTC7A | transmembrane and coiled-coil domain family 2 |
| NM_031412 | HS3ST3B1 | GABA(A) receptor-associated protein like 1 |
| NM_024307 | IFIH1 | glycerophosphodiester phosphodiesterase domain containing 3 |
| NR_024076 | EFS | coxsackie virus and adenovirus receptor pseudogene 3 |
| NM_000784 | ENO3 | cytochrome P450, family 27, subfamily A, polypeptide 1 |
| NM_000362 | TSSK1B | TIMP metallopeptidase inhibitor 3 |
| NM_207391 | SEMA6C | regulator of G protein signaling 9 binding protein |
| NM_001001433 | AMY2A | syntaxin 16 |
| NM_001123392 | TRAM2 | TBC1 domain family, member 3H |
| NM_001037582 | SLC46A3 | stearoyl-CoA desaturase 5 |
| NM_005360 | MYLK4 | v-maf musculoaponeurotic fibrosarcoma oncogene homolog (avian) |
| NM_006483 | FAM84B | dual-specificity tyrosine-(Y)-phosphorylation regulated kinase 1B |
| NM_001042466 | AKAP12 | prosaposin |
| NM_004887 | EGLN3 | chemokine (C-X-C motif) ligand 14 |
| NM_005682 | IL1RN | G protein-coupled receptor 56 |
| NM_001168319 | FBLN5 | endothelin 1 |
| NM_001217 | CHST3 | carbonic anhydrase XI |
| NM_001039705 | WNK2 | trophinin |
| NM_174912 | FTH1 | fatty acid amide hydrolase 2 |
| NR_030775 | AKT1S1 | shisa homolog 4 (Xenopus laevis) |
| NM_017586 | DYSF | chromosome 9 open reading frame 7 |
| NR_003014 | TEAD3 | small nucleolar RNA, H/ACA box 47 |
| NM_004938 | EPB41L4A | death-associated protein kinase 1 |
| NM_015392 | PGAP3 | neural proliferation, differentiation and control, 1 |
| NM_152386 | SNRK | sphingosine-1-phosphate phosphatase 2 |
| NM_006486 | PBX1 | fibulin 1 |
| NM_004390 | EFEMP1 | cathepsin H |
| NM_001128633 | SERPINC1 | RIMS binding protein 3C |
| NM_003629 | RBM4B | phosphoinositide-3-kinase, regulatory subunit 3 (gamma) |
| NM_003186 | TP73 | transgelin |
| NR_033420 | SORT1 | SH3-domain GRB2-like 1 pseudogene 2 |
| NR_026789 | GBP1 | family with sequence similarity 66, member A |
| NM_198455 | TMEM42 | SCO-spondin homolog (Bos taurus) |
| NR_038099_dup1 | MAFA | LIM and senescent cell antigen-like domains 3-like |
| NM_001852 | DNASE2 | collagen, type IX, alpha 2 |
| NM_001171740 | CDH6 | chromosome 3 open reading frame 18 |
| NR_028048 | KRT80 | carnitine O-acetyltransferase |
| NM_130473 | MYL9 | MAP-kinase activating death domain |
| NM_000147 | FUCA1 | fucosidase, alpha-L- 1, tissue |
| NR_027835 | C1orf116 | apolipoprotein L, 3 |
| NM_178548 | TRPM4 | transcription factor AP-2 epsilon (activating enhancer binding protein 2 epsilon) |
| NM_003565 | ZNF385A | unc-51-like kinase 1 (C. elegans) |
| NM_015559 | SNORA74A | SET binding protein 1 |
| NM_182646 | JUP | cytoplasmic polyadenylation element binding protein 2 |
| NM_000699 | BRD3 | amylase, alpha 2A (pancreatic) |
| NM_001166286 | SEMA5A | RGM domain family, member A |
| NM_014553 | TRPV4 | transcription factor CP2-like 1 |
| NM_001006938 | TRIM13 | transcription elongation factor A (SII)-like 6 |
| NM_001085426 | C17orf101 | arylsulfatase A |
| NM_021721 | BCL6 | ADAM metallopeptidase domain 22 |
| NM_138319 | AGPAT3 | proprotein convertase subtilisin/kexin type 6 |
| NM_002570 | ADM | proprotein convertase subtilisin/kexin type 6 |
| NM_001145011 | CBLN3 | chromosome 16 open reading frame 96 |
| NM_015393 | PML | prostate androgen-regulated mucin-like protein 1 |
| NR_002988 | TFAP2E | small nucleolar RNA, H/ACA box 74B |
| NM_206833 | EFNB3 | cortexin 1 |
| NM_001037332 | ELF4 | cytoplasmic FMR1 interacting protein 2 |
| NM_145326 | ARFRP1 | zinc finger protein 493 |
| NM_000891 | LOC100379224 | potassium inwardly-rectifying channel, subfamily J, member 2 |
| NM_014333 | CITED2 | cell adhesion molecule 1 |
| NM_014936 | FLJ44054 | ectonucleotide pyrophosphatase/phosphodiesterase 4 (putative) |
| NM_014767 | TMEM150C | sparc/osteonectin, cwcv and kazal-like domains proteoglycan (testican) 2 |
| NM_001145122 | CLSTN3 | calpain 14 |
| NM_001998 | GFI1 | fibulin 2 |
| NM_017890 | ZNF774 | vacuolar protein sorting 13 homolog B (yeast) |
| NR_002974 | TDO2 | small nucleolar RNA, H/ACA box 42 |
| NM_001040023 | ALG2 | signal-regulatory protein alpha |
| NM_002292 | LRRC37A3 | laminin, beta 2 (laminin S) |
| NM_001003801 | TBC1D13 | SWI/SNF related, matrix associated, actin dependent regulator of chromatin, subfamily d, member 3 |
| NR_003675 | IRF7 | glucuronidase, beta pseudogene 5 |
| NM_001953 | ZMYND10 | thymidine phosphorylase |
| **Genes upregulated** | | |
| **transcript_ID** | **gene** | **desc** |
| NM_001878 | DUOX1 | cellular retinoic acid binding protein 2 |
| NM_001013837 | MRPS28 | MAD1 mitotic arrest deficient-like 1 (yeast) |
| NM_173690 | SNRPD3 | suppressor of cancer cell invasion |
| NM_001010971 | SNORA71D | sterile alpha motif domain containing 13 |
| NM_032294 | COTL1 | calcium/calmodulin-dependent protein kinase kinase 1, alpha |
| NM_001002019 | RPS19BP1 | pseudouridylate synthase 1 |
| NM_001135823 | TNFRSF6B | sarcospan (Kras oncogene-associated gene) |
| NM_001047980 | PCMT1 | neuroblastoma breakpoint family, member 7 |
| NM_206927 | TRMT6 | synaptotagmin-like 2 |
| NM_014882 | C19orf29 | Rho GTPase activating protein 25 |
| NM_001205207 | STK24 | solute carrier family 19 (folate transporter), member 1 |
| NM_017705 | PPM1J | progestin and adipoQ receptor family member V |
| NR_003951 | BAG4 | ADAM metallopeptidase domain 21 pseudogene 1 |
| NM_000265 | PDAP1 | neutrophil cytosolic factor 1 |
| NM_013381 | WDR19 | thyrotropin-releasing hormone degrading enzyme |
| NM_001123375 | LARS2 | histone cluster 2, H3d |
| NM_013363 | PPT1 | procollagen C-endopeptidase enhancer 2 |
| NM_019554 | SNORA68 | S100 calcium binding protein A4 |
| NM_032756 | LINC00346 | 4-hydroxyphenylpyruvate dioxygenase-like |
| NM_153015 | USP24 | transmembrane protein 74 |
| NM_015417 | TMEM147 | sperm flagellar 1 |
| NM_194298 | STK17A | solute carrier family 16, member 9 (monocarboxylic acid transporter 9) |
| NM_001018000 | LTA4H | kazrin, periplakin interacting protein |
| NM_015956 | ABCB7 | mitochondrial ribosomal protein L4 |
| NM_002729 | KLHL17 | hematopoietically expressed homeobox |
| NR_026683 | METTL23 | hypothetical LOC100268168 |
| NR_028374 | THEM4 | small nucleolar RNA, H/ACA box 80B |
| NM_138712 | RDH14 | peroxisome proliferator-activated receptor gamma |
| NM_006176 | ADA | neurogranin (protein kinase C substrate, RC3) |
| NM_002522 | PIN4 | neuronal pentraxin I |
| NM_001197098 | RPF1 | protease, serine, 3 |
| NM_001145364 | ACLY | abhydrolase domain containing 11 |
| NR_024362 | HIST1H4L | family with sequence similarity 86, member A pseudogene |
| NM_004419 | FER | dual specificity phosphatase 5 |
| NM_017522 | MRPL15 | low density lipoprotein receptor-related protein 8, apolipoprotein e receptor |
| NM_025184 | FOXJ3 | EF-hand domain (C-terminal) containing 2 |
| NM_007329 | FAM188A | deleted in malignant brain tumors 1 |
| NM_001203248 | GPN3 | enhancer of zeste homolog 2 (Drosophila) |
| NM_005167 | RFC4 | protein phosphatase, Mg2+/Mn2+ dependent, 1J |
| NM_003177 | TRMT2B | spleen tyrosine kinase |
| NM_016580 | PPP3CB | protocadherin 12 |
| NM_021158 | WDR33 | tribbles homolog 3 (Drosophila) |
| NM_001394 | FEN1 | dual specificity phosphatase 4 |
| NM_018057 | ANKRD39 | solute carrier family 6 (neutral amino acid transporter), member 15 |
| NM_178836 | QTRT1 | phospholipase D family, member 6 |
| NM_018664 | C9orf46 | basic leucine zipper transcription factor, ATF-like 3 |
| NM_017416 | LOC389906 | interleukin 1 receptor accessory protein-like 2 |
| NM_001079675 | GNPDA1 | ets variant 4 |
| NM_145810 | EIF3B | cell division cycle associated 7 |
| NM_005281 | IRAK3 | G protein-coupled receptor 3 |
| NM_005438 | HMGXB4 | FOS-like antigen 1 |
| NM_138969 | SNX9 | short chain dehydrogenase/reductase family 16C, member 5 |
| NM_173575 | TRA2B | serine/threonine kinase 32C |
| NM_001024943 | C2orf48 | argininosuccinate lyase |
| NM_001197294 | FASTKD1 | dihydropyrimidinase-like 3 |
| NR_034128 | MNAT1 | hypothetical LOC440900 |
| NM_001098790 | AAAS | MID1 interacting protein 1 (gastrulation specific G12 homolog (zebrafish)) |
| NR_036475 | METTL6 | Dexi homolog (mouse) pseudogene |
| NM_015175 | PCID2 | neurobeachin-like 2 |
| NM_172240 | RAD51B | POC1 centriolar protein homolog B (Chlamydomonas) |
| NM_001197319 | DNMBP | C-type lectin domain family 2, member D |
| NM_001145319 | RAB23 | plastin 1 |
| NM_001037164 | C1D | ADP-ribosylation factor-like 4A |
| NM_003671 | DCUN1D5 | CDC14 cell division cycle 14 homolog B (S. cerevisiae) |
| NM_000640 | LOC389247 | interleukin 13 receptor, alpha 2 |
| NM_001114121 | FAM60A | CHK1 checkpoint homolog (S. pombe) |
| NM_001432 | GMPPA | epiregulin |
| NM_017955 | DDX21 | cell division cycle associated 4 |
| NM_001006682 | TMEM177 | spindlin family, member 2B |
| NM_001122670 | PSMD5 | phosphorylase kinase, alpha 1 (muscle) |
| NM_017596 | MAPK1 | kinesin family member 21B |
| NM_017816 | MRPS18A | Ly1 antibody reactive homolog (mouse) |
| NM_178858 | SRI | sideroflexin 2 |
| NM_001160130 | LUC7L2 | potassium voltage-gated channel, KQT-like subfamily, member 5 |
| NM_001134876 | CD96 | chromosome 14 open reading frame 80 |
| NM_001080824 | CIAO1 | chromosome 2 open reading frame 89 |
| NM_001645 | C17orf80 | apolipoprotein C-I |
| NM_024712 | GINS3 | engulfment and cell motility 3 |
| NM_018719 | ELOVL6 | cell division cycle associated 7-like |
| NM_001008708 | DIABLO | ChaC, cation transport regulator homolog 2 (E. coli) |
| NM_003104 | TMEM126A | sorbitol dehydrogenase |
| NM_006082 | ZBED2 | tubulin, alpha 1b |
| NM_178177 | PHF5A | nicotinamide nucleotide adenylyltransferase 3 |
| NM_144658 | FAN1 | dedicator of cytokinesis 11 |
| NM_004573 | QPCTL | phospholipase C, beta 2 |
| NM_206836 | MRPL4 | enoyl-CoA delta isomerase 2 |
| NR_024370 | MMS19 | hypothetical LOC440356 |
| NM_024922 | DHX30 | carboxylesterase 3 |
| NM_133367 | PPME1 | progestin and adipoQ receptor family member VIII |
| NM_003218 | TUT1 | telomeric repeat binding factor (NIMA-interacting) 1 |
| NR_027030 | NDUFB3 | hypothetical protein MGC34034 |
| NM_001902 | EIF3I | cystathionase (cystathionine gamma-lyase) |
| NR_036751 | LOC100130932 | heat shock protein 90kDa alpha (cytosolic), class A member 6, pseudogene |
| NM_022370 | SHQ1 | roundabout, axon guidance receptor, homolog 3 (Drosophila) |
| NM_000857 | KCTD1 | guanylate cyclase 1, soluble, beta 3 |
| NM_000187 | KLHL11 | homogentisate 1,2-dioxygenase |
| NM_001013646 | CHCHD2 | chromosome 20 open reading frame 107 |
| NR_027350 | NDUFV2 | miR-17-92 cluster host gene (non-protein coding) |
| NM_001127371 | EIF4H | cell division cycle associated 7-like |
| NM_198527 | KIF1B | HD domain containing 3 |
| NM_001142573 | TATDN3 | IMP (inosine 5'-monophosphate) dehydrogenase 1 |
| NM_015721 | HSPA8 | gem (nuclear organelle) associated protein 4 |
| NM_145247 | SRCRB4D | SWI5-dependent recombination repair 1 |
| NM_013327 | PPP1R2 | parvin, beta |
| NR_000024 | THNSL1 | small nucleolar RNA, C/D box 46 |
| NR_002184 | SNAPC1 | ribosomal RNA processing 7 homolog B (S. cerevisiae) |
| NM_020877 | FAM35B2 | dynein, axonemal, heavy chain 2 |
| NM_080608 | CHCHD4 | chromosome 20 open reading frame 165 |
| NM_001013734 | SENP1 | ret finger protein-like 4B |
| NM_022120 | PP7080 | 3-oxoacid CoA transferase 2 |
| NM_030797 | GYS2 | family with sequence similarity 49, member A |
| NM_001134658 | SUPT16H | solute carrier family 35, member G1 |
| NM_032790 | POLR3B | ORAI calcium release-activated calcium modulator 1 |
| NM_022071 | ANKRD31 | SH2 domain containing 4A |
| NM_145043 | PECR | nei endonuclease VIII-like 2 (E. coli) |
| NR_033249 | HSP90AB1 | glycine cleavage system protein H (aminomethyl carrier) |
| NM_006907 | RPS26 | pyrroline-5-carboxylate reductase 1 |
| NM_174889 | PDE8A | NADH dehydrogenase (ubiquinone) 1 alpha subcomplex, assembly factor 2 |
| NM_006693 | GMEB1 | cleavage and polyadenylation specific factor 4, 30kDa |
| NM_002539 | POLR1E | ornithine decarboxylase 1 |
| NM_053064 | IMPACT | guanine nucleotide binding protein (G protein), gamma 2 |
| NM_015658 | PHLDA2 | nucleolar complex associated 2 homolog (S. cerevisiae) |
| NM_001126130 | HTRA2 | GINS complex subunit 3 (Psf3 homolog) |
| NM_004496 | HMOX2 | forkhead box A1 |
| NM_005797 | NOL6 | myelin protein zero-like 2 |
| NM_004594 | TAF9 | solute carrier family 9 (sodium/hydrogen exchanger), member 5 |
| NM_013300 | CCDC90A | chromosome 12 open reading frame 24 |
| NM_006080 | SPAG16 | sema domain, immunoglobulin domain (Ig), short basic domain, secreted, (semaphorin) 3A |
| NM_134259 | TTC26 | transducin (beta)-like 1, Y-linked |
| NM_018321 | CCDC112 | BRX1, biogenesis of ribosomes, homolog (S. cerevisiae) |
| NM_001013619 | ANAPC7 | aminoglycoside phosphotransferase domain containing 1 |
| NM_001098483 | CELF1 | chromosome 10 open reading frame 125 |
| NM_013332 | CNOT10 | chromosome 7 open reading frame 68 |
| NM_001142295 | TMEM161A | spastic paraplegia 20 (Troyer syndrome) |
| NM_001024599 | SEC61G | histone cluster 2, H2bf |
| NM_001964 | FRAT2 | early growth response 1 |
| NM_012449 | TPCN2 | six transmembrane epithelial antigen of the prostate 1 |
| NM_032852 | C4orf32 | ATG4 autophagy related 4 homolog C (S. cerevisiae) |
| NM_001098832 | GRB2 | family with sequence similarity 104, member A |
| NM_005252 | HMGN2 | FBJ murine osteosarcoma viral oncogene homolog |
| NM_016011 | NAA50 | mitochondrial trans-2-enoyl-CoA reductase |
| NR_024072 | NUP205 | MRS2 magnesium homeostasis factor homolog (S. cerevisiae) pseudogene 2 |
| NM_058179 | AIMP2 | phosphoserine aminotransferase 1 |
| NR_003098 | TCTN2 | small nucleolar RNA host gene 1 (non-protein coding) |
| NM_018440 | PPCDC | phosphoprotein associated with glycosphingolipid microdomains 1 |
| NM_032315 | STT3A | solute carrier family 25, member 33 |
| NM_020685 | CINP | chromosome 3 open reading frame 14 |
| NM_017884 | PTPRG | PIN2/TERF1 interacting, telomerase inhibitor 1 |
| NM_001136498 | DNAJC30 | CDGSH iron sulfur domain 3 |
| NM_024576 | POLR2F | opioid growth factor receptor-like 1 |
| NM_001145971 | SCFD2 | retinol dehydrogenase 13 (all-trans/9-cis) |
| NM_198179 | RPS4X | pyroglutamylated RFamide peptide receptor |
| NM_033438 | ANKRD33B | SLAM family member 9 |
| NM_006331 | GJC1 | EMG1 nucleolar protein homolog (S. cerevisiae) |
| NM_001011666 | EFHC2 | cAMP responsive element binding protein 5 |
| NR_044999 | POLR2J3 | olfactory receptor, family 7, subfamily E, member 12 pseudogene |
| NM_018283 | PLOD3 | nudix (nucleoside diphosphate linked moiety X)-type motif 15 |
| NR_002785 | IMMT | GNAS antisense RNA 1 (non-protein coding) |
| NR_024249 | HAGHL | family with sequence similarity 86, member C2, pseudogene |
| NR_023344 | SHPK | RNA, U6atac small nuclear (U12-dependent splicing) |
| NM_182767 | ANKRD36 | solute carrier family 6 (neutral amino acid transporter), member 15 |
| NM_001080402 | CUL9 | coiled-coil domain containing 61 |
| NM_138809 | DPYSL3 | carboxymethylenebutenolidase homolog (Pseudomonas) |
| NM_003222 | TXNDC11 | transcription factor AP-2 gamma (activating enhancer binding protein 2 gamma) |
| NM_004956 | GNPAT | ets variant 1 |
| NM_001143883_dup1 | AGPAT6 | peptidylprolyl isomerase A (cyclophilin A)-like 4B |
| NM_001143883_dup2 | AGPAT5 | peptidylprolyl isomerase A (cyclophilin A)-like 4B |
| NM_016315 | KCTD15 | GULP, engulfment adaptor PTB domain containing 1 |
| NM_001143831 | ISL2 | glutamate receptor, metabotropic 5 |
| NM_006467 | RBM25 | polymerase (RNA) III (DNA directed) polypeptide G (32kD) |
| NM_005746 | PARP16 | nicotinamide phosphoribosyltransferase |
| NM_173657 | CISD1 | chromosome 3 open reading frame 33 |
| NM_022170 | NELF | eukaryotic translation initiation factor 4H |
| NM_006497 | KLRC3 | hypermethylated in cancer 1 |
| NM_152649 | NFIB | mixed lineage kinase domain-like |
| NM_005053 | RRP7B | RAD23 homolog A (S. cerevisiae) |
| NM_183047 | ASPH | zinc finger, MYND-type containing 8 |
| NM_002703 | RDX | phosphoribosyl pyrophosphate amidotransferase |
| NM_012458 | UBE2D2 | translocase of inner mitochondrial membrane 13 homolog (yeast) |
| NM_015621 | CWC22 | coiled-coil domain containing 69 |
| NM_016271 | SGK196 | ring finger protein 138 |
| NM_005640 | TRUB2 | TAF4b RNA polymerase II, TATA box binding protein (TBP)-associated factor, 105kDa |
| NM_016391 | PIGL | NOP16 nucleolar protein homolog (yeast) |
| NM_015878 | C1orf212 | antizyme inhibitor 1 |
| NM_030780 | STRN4 | solute carrier family 25, member 32 |
| NM_003491 | PAM16 | N(alpha)-acetyltransferase 10, NatA catalytic subunit |
| NM_001039960 | TAB3 | solute carrier family 4, sodium bicarbonate cotransporter, member 8 |
| NM_000902 | NGEF | membrane metallo-endopeptidase |
| NM_014366 | ING1 | guanine nucleotide binding protein-like 3 (nucleolar) |
| NM_025267 | ATF4 | alanyl-tRNA synthetase domain containing 1 |
| NR_027701 | METAP1 | long intergenic non-protein coding RNA 346 |
| NM_018096 | PHB | notchless homolog 1 (Drosophila) |
| NR_028350 | MND1 | prothymosin, alpha pseudogene |
| NM_017659 | RPS3A | glutaminyl-peptide cyclotransferase-like |
| NM_018361 | BOD1 | 1-acylglycerol-3-phosphate O-acyltransferase 5 (lysophosphatidic acid acyltransferase, epsilon) |
| NM_001142405 | TATDN2 | slowmo homolog 1 (Drosophila) |
| NM_001243351 | PLCL2 | negative regulator of ubiquitin-like proteins 1 |
| NM_052903 | ARMC6 | tubulin, gamma complex associated protein 5 |
| NM_001033044 | IGF2BP2 | glutamate-ammonia ligase |
| NM_004282 | C9orf142 | BCL2-associated athanogene 2 |
| NM_006938 | TIMELESS | small nuclear ribonucleoprotein D1 polypeptide 16kDa |
| NM_003095 | TIMM22 | small nuclear ribonucleoprotein polypeptide F |
| NM_001159767 | CDCA7L | basic leucine zipper and W2 domains 2 |
| NR_002454 | MOCS3 | coronin, actin binding protein, 1A pseudogene |
| NM_014251 | STRA13 | solute carrier family 25, member 13 (citrin) |
| NM_001657_dup1 | C18orf19 | amphiregulin |
| NM_000189 | LCE1E | hexokinase 2 |
| NM_153374 | MRPS25 | LysM, putative peptidoglycan-binding, domain containing 2 |
| NM_014154 | C20orf107 | armadillo repeat containing 8 |
| NM_001080420 | SSBP1 | SH3 and multiple ankyrin repeat domains 3 |
| NM_001252 | DCK | CD70 molecule |
| NM_020144 | PPIL3 | poly(A) polymerase beta (testis specific) |
| NM_020226 | RIPK2 | PR domain containing 8 |
| NM_032509 | MSH2 | MAK16 homolog (S. cerevisiae) |
| NR_002818 | SGCE | ring finger protein 126 pseudogene 1 |
| NM_024063 | ANP32A | spermatogenesis associated 5-like 1 |
| NM_015254 | MAP4K4 | kinesin family member 13B |
| NM_138386 | PARL | nuclear assembly factor 1 homolog (S. cerevisiae) |
| NR_024241 | HAL | family with sequence similarity 86, member D, pseudogene |
| NR_026859_dup1 | H2AFZ | family with sequence similarity 7, member A3 |
| NM_152731 | CARS | BEN domain containing 6 |
| NM_020799 | TOMM6 | STAM binding protein-like 1 |
| NM_006230 | AGAP6 | polymerase (DNA directed), delta 2, regulatory subunit 50kDa |
| NM_022838 | C20orf165 | armadillo repeat containing, X-linked 5 |
| NM_001001484 | RPP14 | phosphotriesterase related |
| NM_004775 | C9orf129 | UDP-Gal:betaGlcNAc beta 1,4- galactosyltransferase, polypeptide 6 |
| NM_175886 | ROBO3 | phosphoribosyl pyrophosphate synthetase 1-like 1 |
| NM_000270 | RAC3 | purine nucleoside phosphorylase |
| NM_004278 | PTCD3 | phosphatidylinositol glycan anchor biosynthesis, class L |
| NM_014880 | DBNL | CD302 molecule |
| NM_020704 | GXYLT1 | family with sequence similarity 40, member B |
| NR_024275 | MIPEP | hypothetical LOC151162 |
| NR_024529 | SLC25A12 | ribosomal protein L23a pseudogene 7 |
| NM_024590 | C22orf40 | arylsulfatase family, member J |
| NM_145041 | UBTF | transmembrane protein 106A |
| NM_207342 | TPK1 | STEAP family member 1B |
| NM_020243 | VAV3 | translocase of outer mitochondrial membrane 22 homolog (yeast) |
| NM_006430 | DAP3 | chaperonin containing TCP1, subunit 4 (delta) |
| NM_006278 | TOMM34 | ST3 beta-galactoside alpha-2,3-sialyltransferase 4 |
| NM_001130528 | TMEM136 | sperm associated antigen 9 |
| NM_018319 | APOO | tyrosyl-DNA phosphodiesterase 1 |
| NM_004787 | TAS2R43 | slit homolog 2 (Drosophila) |
| NM_001166692 | CCDC75 | chromosome 11 open reading frame 91 |
| NM_004728 | ESF1 | DEAD (Asp-Glu-Ala-Asp) box polypeptide 21 |
| NM_201400 | HACE1 | family with sequence similarity 86, member A |
| NM_014285 | GPATCH1 | exosome component 2 |
| NM_002237 | LUC7L | potassium voltage-gated channel, subfamily G, member 1 |
| NR_024253 | HAPLN3 | family with sequence similarity 86, member E, pseudogene |
| NM_019042 | RPS2 | pseudouridylate synthase 7 homolog (S. cerevisiae) |
| NM_023077 | SP4 | Sel1 repeat containing 1 |
| NM_014638 | QRSL1 | phospholipase C, eta 2 |
| NM_017819 | SEPHS1 | RNA (guanine-9-) methyltransferase domain containing 1 |
| NM_001205281 | MFN2 | serine/threonine-protein phosphatase 5-like |
| NM_001025247 | TSC22D4 | TAF5-like RNA polymerase II, p300/CBP-associated factor (PCAF)-associated factor, 65kDa |
| NM_005795 | COQ2 | calcitonin receptor-like |
| NM_000530 | NOL12 | myelin protein zero |
| NM_001074 | ZNF710 | UDP glucuronosyltransferase 2 family, polypeptide B7 |
| NM_001040285 | PPIH | PAP associated domain containing 5 |
| NM_014165 | PDHA1 | NADH dehydrogenase (ubiquinone) 1 alpha subcomplex, assembly factor 4 |
| NM_004741 | PICALM | nucleolar and coiled-body phosphoprotein 1 |
| NM_006916 | ALG14 | ribulose-5-phosphate-3-epimerase |
| NM_078629 | NUP88 | male-specific lethal 3 homolog (Drosophila) |
| NM_014681 | FAM120A | DEAH (Asp-Glu-Ala-His) box polypeptide 34 |
| NM_012403 | C14orf93 | acidic (leucine-rich) nuclear phosphoprotein 32 family, member C |
| NM_000328 | SKA2 | retinitis pigmentosa GTPase regulator |
| NM_005504 | CACNB1 | branched chain amino-acid transaminase 1, cytosolic |
| NM_001657 | C17orf58 | amphiregulin |
| NM_017544 | PHAX | NFKB repressing factor |
| NM_001184743 | AEBP2 | piggyBac transposable element derived 1 |
| NR_026878 | NDUFB2 | hypothetical protein MGC12982 |
| NM_014473 | FAM136A | DIM1 dimethyladenosine transferase 1 homolog (S. cerevisiae) |
| NM_000137 | GPR3 | fumarylacetoacetate hydrolase (fumarylacetoacetase) |
| NM_001010905 | CLPX | chromosome 6 open reading frame 58 |
| NM_018464 | DNAJC2 | CDGSH iron sulfur domain 1 |
| NM_002863 | RPS27 | phosphorylase, glycogen, liver |
| NM_005845 | ATL2 | ATP-binding cassette, sub-family C (CFTR/MRP), member 4 |
| NM_003703 | PIGH | NOP14 nucleolar protein homolog (yeast) |
| NM_024678 | PCDH12 | asparaginyl-tRNA synthetase 2, mitochondrial (putative) |
| NR_027669 | SGPP1 | ring finger protein 170 |
| NM_198867 | C12orf39 | alkB, alkylation repair homolog 6 (E. coli) |
| NM_014038 | CDCA7 | basic leucine zipper and W2 domains 2 |
| NM_001103161 | SRSF7 | SH2 domain containing 5 |
| NR_002327 | TDRKH | small nucleolar RNA, H/ACA box 10 |
| NM_018290 | PSMA5 | phosphoglucomutase 2 |
| NM_032194 | SIX1 | ribosome production factor 2 homolog (S. cerevisiae) |
| NM_001017928 | CUL3 | coiled-coil domain containing 58 |
| NM_006636 | OR7E12P | methylenetetrahydrofolate dehydrogenase (NADP+ dependent) 2, Metheny tetrahydrofolate cyclohydrolase |
| NR_034077 | METTL9 | hypothetical LOC100499177 |
| NM_152400 | CLASRP | chromosome 4 open reading frame 32 |
| NM_001199344 | ALG6 | ribosomal protein L17 |
| NM_001634 | C12orf43 | adenosylmethionine decarboxylase 1 |
| NM_017906 | PPIA | PAK1 interacting protein 1 |
| NM_002082 | ISCU | G protein-coupled receptor kinase 6 |
| NR_033417 | JTB | general transcription factor IIH, polypeptide 2B |
| NM_001172574 | ZBTB24 | microcephalin 1 |
| NR_037676 | CD99 | chromosome 14 open reading frame 82 |
| NM_173467 | TSPAN4 | malonyl CoA: ACP acyltransferase (mitochondrial) |
| NM_018360 | ZDHHC8 | taxilin gamma |
| NM_007080 | MRPL48 | LSM6 homolog, U6 small nuclear RNA associated (S. cerevisiae) |
| NM_033169 | C8orf38 | beta-1,3-N-acetylgalactosaminyltransferase 1 (globoside blood group) |
| NR_002957 | TEAD4 | small nucleolar RNA, H/ACA box 15 |
| NM_019844 | TARDBP | solute carrier organic anion transporter family, member 1B3 |

| **Table S2, Antibodies list for this study** | | | |
| --- | --- | --- | --- |
| **Antibodies Name** | **Resource** | **Catalogue number** |  |
| LMP1 | DAKO | M0897 |  |
| OGDH | Cell signaling | #13407S |  |
| HK2 | Cell signaling | #2867 |  |
| PFKL | Cell signaling | #8175 |  |
| PFKFB3 | Cell signaling | #13123 |  |
| PGAM | Cell signaling | #12098 |  |
| Enolase2 | Cell signaling | #8171 |  |
| PDHK1 | Cell signaling | #3820 |  |
| β-actin | Santa Cruz | SC-47778 |  |
| E-Cadherin | Santa Cruz | SC-21791 |  |
| DSC2 | Life technology | 32-6200 |  |
| N-Cadherin | Santa Cruz | SC-7939 |  |
| Vimentin | Cell signaling | #3932 |  |
| Snail | Cell signaling | #3879 |  |
| Slug | Cell signaling | #9585 |  |
| Twist | Santa Cruz | SC-15393 |  |
| pAKT Ser473 (for IHC) | Abcam | ab81283 |  |
| pAKT Ser473 | Cell signaling | #9271S |  |
| AKT | Cell signaling | #4691 |  |
| pRictor Thr1135 | Cell signaling | #3806S |  |
| Rictor | Santa Cruz | SC-99004 |  |
| Vinculin | Sigma | V9131 |  |
| pPDHE1α Ser293 | Abcam | Ab92696 |  |
| PDHE1α | Santa Cruz | SC-377092 |  |
| mTOR | Cell signaling | 2972S |  |
| GAPDH | Proteintech | 10494-1-AP |  |
| COX Ⅳ | Cell signaling | #4805P |  |
| Histone H3 | Cell signaling | #3638P |  |
| HA | Covance | MMS-101R |  |
| H3K9Ac | Cell signaling | #9649 |  |
| H4Ac (pan) | Santa Cruz | SC-34263 |  |
| Histone H4 | Santa Cruz | SC-25260 |  |
| IGF-1 | Abcam | ab9572 |  |
| pIGF1R Tyr1161 | Abcam | ab39398 |  |
| IGF1R | Santa Cruz | sc-462 |  |
| anti-Rabbit 2^nd^ | Cell signaling | #7074 |  |
| anti-Mouse 2^nd^ | Cell signaling | #7076 |  |
| Alexa Fluro 488 rabbit anti Mouse | Molecular Probes | A11059 |  |
| Alexa Fluro 555 goat anti Rabbit | Molecular Probes | A21428 |  |

| **Table S3, Primers list for this study** | | | |
| --- | --- | --- | --- |
| **Primers for metabolic genes (Roche system)** | | | |
| **Gene name** | **Probe#** | **Forward primer** | **Reverse primer** |
| HK1 | #25 | cacctgtgaggttggactca | ccaccatctccacgttcttc |
| HK2 | #22 | tccttccctgaaccttttcc | cagatttcaagagacatgacattagc |
| PGI | #1 | gggaaaatcgacggagga | gggcgatttccttcaaagac |
| PFKM | #69 | agcagtaaagacgttaagggtatca | tcagacagccagcaagtagtg |
| PGK1 | #3 | gagccagttgctgtagaactca | cttctgggcctacacagtcc |
| PGAM1 | #51 | aggatcagctccttgacctct | aacaaggcaggaagggattc |
| Enolase1 | #1 | tcccaacatcctggagaataa | atgccgatgaccaccttatc |
| PKM | #1 | ccttcattcagacccagcag | ctccactgatcgggaagc |
| NQR | #60 | tctgacaccttatgcactgaaga | aagaagataattggaacgcaaatc |
| COX | #4 | gtgtttctgttgcgggaag | gagaagaacgtagccgcttg |
| CS | #6 | ggctgctggtaactggacata | cctctttgcccactcttttg |
| β-actin | #9 | agagctacgagctgcctgac | cgtggatgccacaggact |
|  |  |  |  |
| **Primers for cloning** | | | |
| PDHE1α | WT | taagcaggtaccatgaggaagatgctc | taagcactcgagttaactgactgacttaaa |
|  | S to A | gaattcacacagtatggctgaccctggag | gaattcctccagggtcagccatactgtgt |
|  | S to D | gaattcacacagtatggatgaccctggag | gaattcctccagggtcatccatactgtgt |
| PDHK1 | WT | taagcaggtaccatgaggctggcgcggct | taagcactcgagctaggcactgcggaa |
|  | S to A | gaattcgacctcgtgttgaggcctcccgcgcag | gaattcctgcgcgggaggcctcaacacgaggtc |
|  |  |  |  |
| **Primers for ChIP assay Snail promoter (SYBR Green system)** | | | |
| **Region** | **Primer#** | **Forward primer** | **Reverse primer** |
| -4000 | #1 | gaaaatgcaggtgtctattc | aaggctggtataactgccg |
| -3000 | #2 | tacagtagaaagactaacaat | aggggaggcaagaacaggc |
| -2000 | #3 | atagtgaccaaatttttc | tcagcctcgtttagtgaag |
| -1000 | #4 | ggcccagcacatctgacc | cgagggaagaagtggcaa |
| -500 | #5 | cgtcaatgccacgctct | ccgggacacctgaccttc |
| -250 | #6 | ccgccacgcggcgcgagc | cccctttgtcacctcc |
| 0 | #7 | acttaagggagttggcgg | cactggggtcgccgattc |
| 500 | #8 | gagatgtgtgtgaggag | ccctctcctaagtccca |
| 1000 | #9 | agagctgacctccctgca | agagactgaagtagagga |
| 2000 | #10 | agcatatgttttagagagt | ttcagtgggggtggggaa |
